# Functional reorganization of brain regions supporting artificial grammar learning across the first half year of life

**DOI:** 10.1371/journal.pbio.3002610

**Published:** 2024-10-22

**Authors:** Lin Cai, Takeshi Arimitsu, Naomi Shinohara, Takao Takahashi, Yoko Hakuno, Masahiro Hata, Ei-ichi Hoshino, Stuart K. Watson, Simon W. Townsend, Jutta L. Mueller, Yasuyo Minagawa

**Affiliations:** 1 Department of Electronics and Electrical Engineering, Keio University, Yokohama, Japan; 2 Global Research Center for Logic and Sensitivity, Global Research Institute, Keio University, Tokyo, Japan; 3 Department of Pediatrics, Keio University School of Medicine, Shinjuku, Tokyo, Japan; 4 Department of Comparative Language Science, University of Zürich, Zurich, Switzerland; 5 Department of Evolutionary Biology and Environmental Studies, University of Zürich, Switzerland; 6 Center for the Interdisciplinary Study of Language Evolution, University of Zurich, Zürich, Switzerland; 7 Department of Evolutionary Anthropology, University of Zurich, Zürich, Switzerland; 8 Department of Psychology, University of Warwick, Coventry, United Kingdom; 9 Department of Linguistics, University of Vienna, Vienna, Austria; 10 Vienna Cognitive Science Research HUB, Vienna, Austria; 11 Department of Psychology, Faculty of Letters, Keio University, Yokohama, Japan; 12 Human Biology-Microbiome-Quantum Research Center, Keio University, Tokyo, Japan; ICREA, University of Barcelona, SPAIN

## Abstract

Pre-babbling infants can track nonadjacent dependencies (NADs) in the auditory domain. While this forms a crucial prerequisite for language acquisition, the neurodevelopmental origins of this ability remain unknown. We applied functional near-infrared spectroscopy in neonates and 6- to 7-month-old infants to investigate the neural substrate supporting NAD learning and detection using tone sequences in an artificial grammar learning paradigm. Detection of NADs was indicated by left prefrontal activation in neonates while by left supramarginal gyrus (SMG), superior temporal gyrus (STG), and inferior frontal gyrus activation in 6- to 7-month-olds. Functional connectivity analyses further indicated that the neonate activation pattern during the test phase benefited from a brain network consisting of prefrontal regions, left SMG and STG during the rest and learning phases. These findings suggest a left-hemispheric learning-related functional brain network may emerge at birth and serve as the foundation for the later engagement of these regions for NAD detection, thus, providing a neural basis for language acquisition.

## Abstract 2

Infants have impressive auditory learning capabilities, even from the newborn stage. This study provides neuroimaging evidence that both newborns and 6–7-month-olds already learn and detect grammar-like rules, and that the underlying brain networks are reorganized and more adult-like after the first half year.

## Introduction

Humans are born with sophisticated auditory abilities, possibly shaped by prenatal experience and a relatively mature auditory system [1,2]. Studies with newborn infants have thus demonstrated impressive abilities in the auditory domain including discrimination of stimuli based on various auditory features [3,4], auditory learning of novel sounds [5,6], and computation of more complex sound sequences [7–10]. The latter is of particular relevance for language acquisition, as human syntax relies on the ability to decode a hierarchical structure from sequentially organized auditory input. The sequential auditory input can involve more or less complex dependency patterns ranging from simple adjacent dependencies, i.e., the relation of 2 consecutive stimuli, to multiple embedded nonadjacent dependencies. Nonadjacent dependencies (NADs) are important for language because they allow the meaningful relation of elements across a distance, enabling the formation of complex and hierarchical sentence structures [11]. For example, the sentence “The baby smiles” that contains an NAD between the verb suffix–s and the noun, can be extended to “The baby who is sitting on her mother’s lap smiles” that contains a further NAD between the suffix–ing and the auxiliary “is,” creating a nested structure of NADs. In order to analyze such sentences, the dependent elements have to be stored and retrieved across variable distances. The basic computational mechanisms underlying the ability to detect NADs have been studied both using linguistic as well as nonlinguistic materials [12–15]. The ability to learn NADs seems to be present from early on, both when encoded in speech [16] and when encoded in computationally simpler sine-tone sequences [12,13]. Even nonhuman animals seem to be able to detect NADs in some cases [17,18] suggesting that the basic ability may not be unique to humans and constitutes an important computational basis for language which is present early in development and potentially rooted in our primate ancestors. What is not known, though, is (i) from which age onwards the ability to detect NADs is present in humans; and (ii) which brain areas are involved during learning and detection of NADs in those early developmental stages.

The ability to learn NADs in the auditory domain and its developmental trajectory have been subject to intense investigations in infants and young children [11,19]. Previous behavioral investigations have shown that a sensitivity to NADs, embedded in natural or artificial language, appears after the first year of life [20–23] (around 15 to 19 months), and the ability to discriminate nonadjacent repetitions (e.g., ABA) from other patterns (e.g., ABB or ABC) is present in 5- to 7-month-old infants [24,25]. However, such findings may be constrained by the restrictions of behavioral measures, i.e., the dependence of those measures on overtly observable responses. Electrophysiological methods, for example, present a more fine-grained approach toward directly probing the recognition of NADs, and have correspondingly validated infants’ earlier sensitivity to NADs [15,26]. For example, an event-related potential (ERP) study using the familiarization-test paradigm suggests that 4-month-old monolingual infants can passively learn NADs from a foreign language, indicated by a late positive ERP effect in response to NAD violations after learning [15]. Several other electrophysiological studies attested NAD learning before the first birthday [12,26,27]. While the learning of adjacent dependencies has been demonstrated already in newborns [9,10], there is no evidence so far for the learning of NADs in newborns.

Unfortunately, electrophysiological evidence alone cannot provide precise information concerning the question of which brain regions are involved in NAD learning. Recent functional magnetic resonance imaging (fMRI) advances in adults reveal that Broca’s region is involved in the processing of NADs [28–30]. Similarly, van der Kant and colleagues [13] found, using functional near-infrared spectroscopy (fNIRS), that NAD violation detection was subserved by a left-hemispheric temporo-fronto-parietal network for linguistic stimuli in 2-year-olds. On the other hand, 3-year-olds, but not 2-year-olds were able to learn NADs consisting of nonlinguistic tone stimuli engaging a bilateral temporo-parietal network. However, there is no evidence that NAD learning in tone stimuli develops later than in language stimuli as demonstrated by a recent ERP evidence showing that infants who were only 5 months of age can track embedded NAD structures between simple sine tone stimuli [12]. A study focusing on the specific role of prosodic cues for NAD learning in 9-month-old infants revealed the contribution of frontotemporal brain regions in the more difficult, monotonous condition and the engagement of temporal brain areas in the presence of prosodic cues [14]. Yet, this study leaves unclear whether the infants learned NADs between 2 specific elements or only positional regularities. Together, these findings indicate that NAD learning may be present early in development and that neural networks that include classic language areas are involved when correct and incorrect NADs are successfully discriminated from early childhood onwards, at least in the case of linguistic stimuli. However, it is not known whether similar neural networks support NAD learning and detection from birth and how they develop across the first half year of life.

A growing body of research demonstrates that neonates possess an adult-like ventral pathway for language, which connects the anterior temporal lobe with the ventrolateral prefrontal cortex by the extreme capsule [31]. Likewise, the part of the dorsal pathway connecting the temporal cortex to the premotor cortex is also already present at birth. These structural connections may serve as a neural basis for rule learning [31], maternal speech perception [32], and phonological learning [5] in neonates. By contrast, the part of the dorsal pathway connecting the temporal cortex to Broca’s area develops much later, but it catches up during the first postnatal months [33]. Note that this connection has been argued to be involved in parsing more complex syntactic structures during childhood [31,34]. Taking into account both the early functionality of the prefrontal cortex and its relative immaturity, a very recent review [35] proposed that infants learn actively from their environment early on and that this is supported by their (anatomically) immature prefrontal cortex. Taken together, these studies reveal that an intriguing functional brain network consisting of several distributed key brain regions for language processing has emerged since birth. Further, they suggest that the remarkable learning ability of NADs in older infants reported above could be supported by the ventral and dorsal pathways despite the relative immaturity of the prefrontal cortex (and its dorsal connection to posterior brain areas).

**Fig 1:**
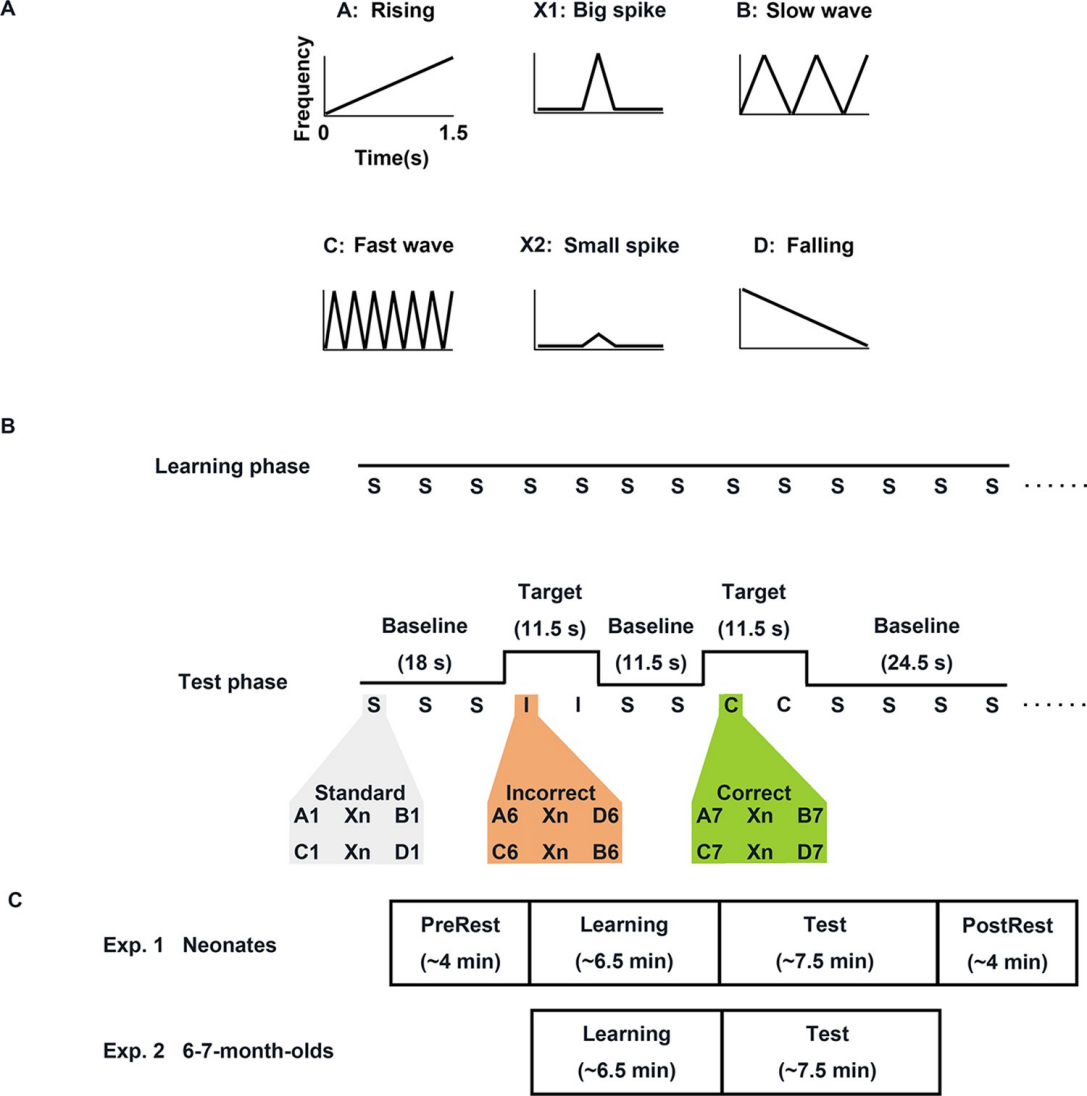
Stimuli and experimental design. **(A)** The pitch contours of 6 acoustic categories. **(B)** Experimental design. In the Learning phase, standard triplets were repeated 60 times. In the Test phase, 2 types of target trials (Correct and Incorrect conditions) were separated by a jittering baseline condition in which the same standard triplets with the Learning phase were presented. **(C)** Measurement phases for 2 experiments.

Because NAD learning seems to be present early in development and distributed across various vertebrate species and also relevant for core processes of language, as outlined above, we hypothesize that (i) humans are born with the ability to track NADs; and (ii) the brain regions that support this process may include areas that are also involved in early language processing including left hemispheric fronto-temporal networks. In the current study, we examined the neurodevelopmental origins of NAD learning and detection using nonlinguistic tone sequences in order to further understand the ontogenesis of human language. We chose nonlinguistic stimuli as we aimed to test the ability to learn NADs from auditory input in an experience-independent manner and as we further wanted to be able to compare the results to previous findings in nonhuman animals [17]. Furthermore, it is well established from previous studies that sequential learning in infancy works with both linguistic and nonlinguistic auditory stimuli [12,36,37]. The NADs in the current study were realized as dependencies between nonadjacent tone categories mimicking syntactic relations between word categories, as for example between nouns and verbs with agreement marking. We adopted the fNIRS technique to test neonates (1 to 5 days old) in Experiment 1 and infants (6 to 7 months old) in Experiment 2 using an auditory artificial grammar learning paradigm (Fig 1B). We included neonates to test neural processes involved in learning and detection of NADs, as outlined above, at birth. Infants aged 6 to 7 months were chosen because a more stable global neural system for language processing appears to be established at this age compared to younger ages, as judged by their resting-state connectivity from the language areas [38]. Additionally, we needed an infant group older than 3 to 4 months who would show a reliable response to detect NADs [15,26] to compare the neonate group. Note that our experimental design with the chosen participant groups allowed the assessment of brain regions during NAD detection in both age groups, while the assessment of regions involved during learning could be assessed in neonates.

In both experiments, the stimuli were composed of a series of perceptually simple tone sequences, each of which comprised 3 frequency-modulated sine tones from 6 categories of pitch contour (A, B, C, D, X1, and X2; see Fig 1A). Each sine tone category had various pitch variants which were used to avoid stimulus-specific learning. In the learning (Learning) phase, participants were exposed to 60 standard tone triplets conforming to NAD rules (e.g., AXB or CXD grammar) for approximately 6 min. Subsequently, during the test (Test) phase, they were presented with the familiar standard sequences and sequences comprised of novel pitch variants of the acoustic categories heard during the Learning phase arranged into either “Correct” triplets (e.g., AXB or CXD) or “Incorrect” triplets (e.g., AXD or CXB), thereby testing whether individuals were able to generalize the NAD rules from the Learning phase and detect violations. In Experiment 1, we additionally recorded the hemodynamic activities of neonates during the pre-task resting-state (Pre-Rest) phase, the Learning phase, and the post-task resting-state (Post-Rest) phase. Pre-Rest and Post-Rest phases were inserted before and after Learning and Test phases, respectively. The 2 resting-state phases allowed us to examine changes in functional connectivity (FC) from Pre-Rest to Learning phases or from Pre-Rest to Post-Rest phases, as well as the relationship between the FC changes and the activation to NAD violations during the Test phase (Fig 1C). In Experiment 2, we exposed 6- to 7-month-olds to Learning and Test phases without resting-state phases but we performed fNIRS scans only during the Test phase (Fig 1C). The decision to limit scan time in this experiment was based on the increased level of physical activity and thus potentially decreased compliance with the procedure in this age group.

Thus, the first aim of Experiment 1 was to reveal whether human neonates can extract NAD relations and generalize them to novel tone sequences, which would be indicated by a greater neural response to incorrect sequences than to correct ones during the Test phase. The second aim of Experiment 1 was to identify, in the case of successful NAD violation detection, the brain networks underlying NAD learning at birth by examining FC changes (Learning minus Pre-Rest or Post-Rest minus Pre-Rest) and their correlations to cerebral responses to learned NAD relations as measured during the Test phase. The aim of Experiment 2 was to examine what brain networks underlie the successful detection of NADs in 6- to 7-month-old infants. By combining these 2 experiments, we could shed light on the emergence and development of the functional brain network underlying the detection of NADs across the first half year of life.

## Results

### Experiment 1

**Fig 2:**
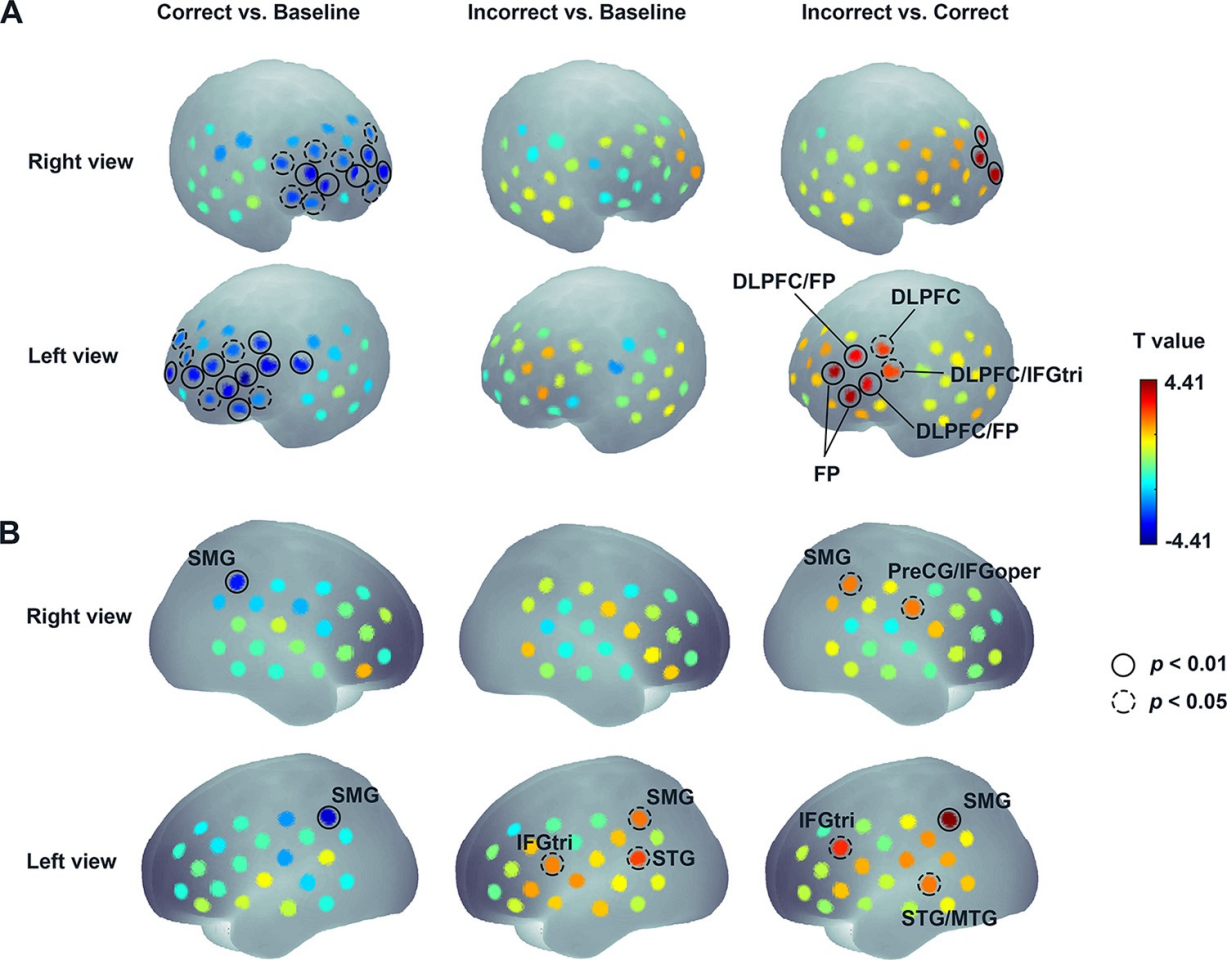
The statistically significant effects in the channel-by-channel permutation *t* test analysis. **(A)** Experiment 1: neonates. **(B)** Experiment 2: 6- to 7-month-olds. Left, middle, and right panels show brain regions that were activated significantly for 3 condition contrasts of Correct vs. Baseline, Incorrect vs. Baseline, and Incorrect vs. Correct, respectively. The circles denote significant channels (solid circles: permutation *p* < 0.01; dotted circles: permutation *p* < 0.05). The data underlying this figure can be found at https://osf.io/84yu9/.

As illustrated in Fig 2A (left and middle), mean hemodynamic response within the 5-s time window to the Correct and Incorrect conditions as indicated by oxygenated hemoglobin (HbO) changes was compared with that of the baseline period for each channel (Ch). Permutation *t* tests (S1 Table) confirmed that neonates showed significantly decreased activation for the Correct condition compared to baseline. Significant activation was chiefly found in the prefrontal regions including Ch 3, Ch 25–26, Ch 28–39, and Ch 41–43 (Fig 2A, left). On the other hand, although no significant differences were observed for the Incorrect versus baseline (Fig 2A, middle), a comparison between the Incorrect and Correct conditions (Fig 2A, right) revealed a greater activation dominantly in the left hemisphere. These significant brain regions included left (L) frontal pole (L-FP) (Ch 30: t = 4.05, *p* = 0.001; Ch 35: t = 4.04, *p* = 0.001), L dorsolateral prefrontal cortex (L-DLPFC)/L-FP (Ch 34: t = 3.49, *p* = 0.003; Ch 39: t = 3.32, *p* = 0.003), L-DLPFC/L triangular part of inferior frontal gyrus (L-IFGtri) (Ch 38: t = 2.71, *p* = 0.012), and L-DLPFC (Ch 43: t = 2.84, *p* = 0.008). (See S1 Table for a summary of statistical results and S2 Fig for the grand averaged time courses of the hemodynamic responses for 2 experimental conditions.)

We did not find any significant results when using deoxygenated hemoglobin (HbR) changes as an index. All HbR results are summarized in S2 Table.

**Fig 3:**
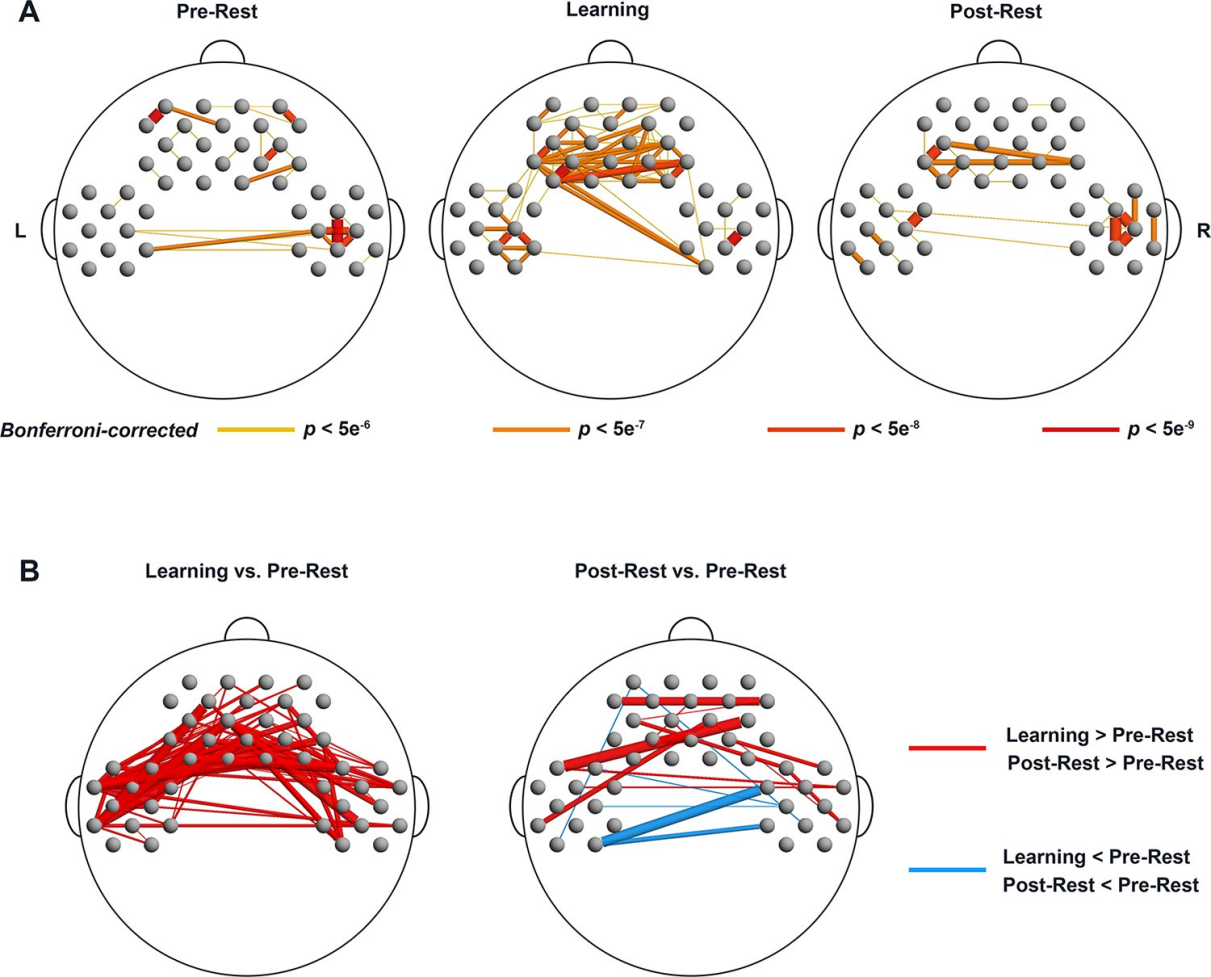
The FC patterns in Experiment 1: Neonates. **(A)** The most significantly increased FCs for 3 phases against a respective zero baseline. Different colors and line thickness indicate different thresholds. Both darker color and thicker line indicate the more stringent threshold. **(B)** The FCs with significant changes for the contrasts of Learning phase vs. Pre-Rest phase, and Post-Rest phase vs. Pre-Rest phase. The data underlying this figure can be found at https://osf.io/84yu9/. FC, functional connectivity.

Experiment 1 further explored the FC patterns during the Pre-Rest phase, the Learning phase, and the Post-Rest phase. For each of the 3 phases alone, there were a large number of significantly stronger FCs relative to a respective zero baseline, with more than 445 significant FCs (Bonferroni corrected *p* < 0.05, S4 Fig). To further explore the locations of the most significant FCs in the brain, we set stringent thresholds at 4 levels (Bonferroni corrected *p* < 5e-6, *p* < 5e-7, *p* < 5e-8, and *p* < 5e-9) as indicated in Fig 3A. It showed that the Learning phase exhibits a larger number of significant FCs compared to the other 2 resting-state phases. Particularly, FCs connecting frontal and temporal regions emerged during the learning phase, which were absent during the other 2 resting-state phases. Furthermore, we compared FC differences between the Learning and Pre-Rest phases, as well as between the Post-Rest and Pre-Rest phases. For the first comparison (Learning minus Pre-Rest), we found 96 significantly increased FCs (uncorrected *p* < 0.05; Fig 3B, left). For the second comparison, we found 13 significantly increased FCs and 6 significantly decreased FCs (uncorrected *p* < 0.05; Fig 3B, right).

**Fig 4:**
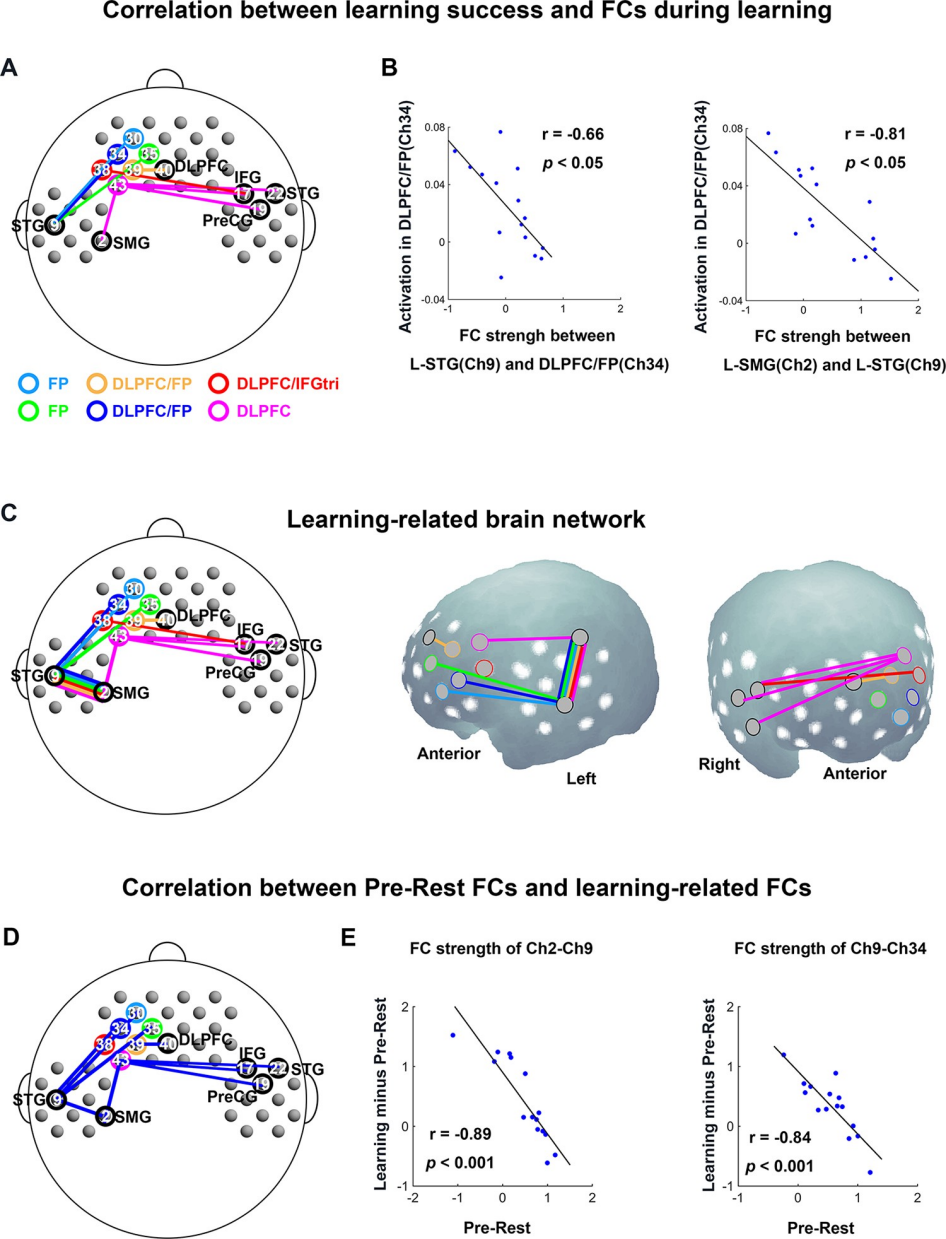
Functional brain network underlying NAD learning in Experiment 1: Neonates. **(A)** Relations between prefrontal activation during the Test phase (learning success) and FCs with significant changes from the Pre-Rest phase to the Learning phase (FCs during learning). Seed channels are indicated by circles with 6 different colors and non-seed channels indicated by black circles. The lines with 6 different colors represent FCs displaying significantly negative correlation with activations in seed channels. **(B)** The negative correlation between activation in Ch 34 and FC strength (Ch 9–Ch 34; Ch 2–Ch 9). The blue dots represent data from 15 neonates. **(C)** 2D and 3D maps of the learning-related brain network, where the strength of all FCs was negatively correlated with activation in the 6 seed channels. **(D)** Relations between FC strength during the Pre-Rest phase and FC strength changes from the Pre-Rest phase to the Learning phase (Learning minus Pre-Rest). Blue lines indicate negative correlations. **(E)** The scatter plots from 2 canonical negative correlations in Fig 4D. The blue dots represent data from 15 neonates. The data underlying this figure can be found at https://osf.io/84yu9/. FC, functional connectivity; NAD, nonadjacent dependency.

To examine how FCs during NAD learning relate to actual learning success (i.e., detection of NAD violations), we analyzed the correlations between the strength of FCs with significant changes during the Learning phase (i.e., Learning minus Pre-Rest) and the degree of activation in those 6 prefrontal channels that were significantly activated during the Test phase (Ch 30, Ch 34, Ch 35, Ch 38, Ch 39, Ch 43, termed seed channels from here onwards). Thus, FCs connecting any one of those 6 seed channels were examined. Notably, as illustrated in Fig 4A, the strength of 9 FCs during the Learning phase (Ch 30–Ch 9, r = −0.52; Ch 34–Ch 9, r = −0.66; Ch 35–Ch 9, r = −0.55; Ch 38–Ch 17, r = −0.54; Ch 39–Ch 40, r = −0.68; Ch 43–Ch 2, r = −0.57; Ch 43–Ch 17, r = −0.56; Ch 43–Ch 19, r = −0.60; Ch 43–Ch 22, r = −0.56; all ps < 0.05) were negatively correlated with the activations in seed channels (A canonical negative relationship, see Fig 4B, left). From the perspective of anatomy, these FCs mainly connected prefrontal regions to L supramarginal gyrus (L-SMG) and L superior temporal gyrus (L-STG), as well as right (R)-IFG, R precentral gyrus (R-PreCG), and R-STG. To further examine whether these 9 FCs during the Learning phase (Fig 4A) and other FCs within these channels form a learning-related brain network together, we analyzed the correlations between the FC strength of any 2 non-seed channels involved in the 9 FCs (i.e., L-STG, L-SMG, R-IFG, R-STG, R-PreCG, DLPFC) and the activation degree of 6 prefrontal seed channels. As shown in Fig 4B (right), the strength of FC linking L-SMG (Ch 2) and L-STG (Ch 9) was significantly negatively related to the activation degree of all 6 seed channels (r values: Ch 30, −0.68; Ch 34, −0.81; Ch 35, −0.70; Ch 38, −0.58; Ch 39, 0.67; Ch 43, −0.66, ps < 0.05). Finally, all 10 (9+1) intrinsic FCs showing negative correlations with activation in 6 prefrontal channels formed a learning-related brain network as shown in Fig 4C, which may be responsible for NAD learning.

To better understand the reason why the learning-related brain network with 10 intrinsic FCs was negatively related to the prefrontal activation found for NAD violation detection, we explored the relationship between FC strength during the Pre-Rest and Learning phases for the 10 intrinsic FCs. We found 9 (out of 10) negative correlations between FC strength during Pre-Rest and Learning minus Pre-Rest to be significant (r values: Ch 2–Ch 9, −0.89; Ch 9–Ch 30, −0.71; Ch 9–Ch 34, −0.84; Ch 9–Ch 35, −0.80; Ch 39–Ch 40, −0.84; Ch 2–Ch 43, −0.82; Ch 17–Ch 43, −0.58; Ch 19–Ch 43, −0.68; Ch 22–Ch 43, −0.76, ps < 0.05) (Fig 4D and 4E). This meant that while most neonates showed stronger FCs during the Learning phase compared to the Pre-rest phase (S5 Fig), neonates with stronger FC as a default state (i.e., Pre-Rest) tended to show less increment of FC from Pre-Rest to Learning phases. Likewise, a negative correlation between FC strength for Pre-Rest and that for Post-Rest minus Pre-Rest (Ch 34–Ch 17) was confirmed, which revealed a similar link between default-state and later observed changes (S6 Fig).

### Experiment 2

As illustrated in Fig 2B (left and middle), the mean hemodynamic response within the 5-s time window to the Correct and Incorrect conditions as indicated by HbO changes was compared with that of the baseline period for each channel. Permutation *t* tests (S3 Table) revealed significantly decreased responses to the Correct condition relative to the baseline in L-SMG and R-SMG (Ch 4: t = −3.71, *p* = 0.002; Ch 23: t = −3.13, *p* = 0.003). For the Incorrect condition versus baseline, significantly increased responses were exclusively observed in the left hemisphere including L-SMG (Ch 4: t = 2.32, *p* = 0.031), L-IFGtri (Ch 11: t = 2.17, *p* = 0.045), and L-STG (Ch 13: t = 2.75, *p* = 0.013). Notably, comparisons between the 2 conditions (Fig 2B, right) showed greater responses to the Incorrect condition relative to the Correct condition, particularly in the L-SMG (Ch 4: t = 4.41, *p* = 0.001), L-IFGtri (Ch 6: t = 2.95, *p* = 0.011), and L-STG/L middle temporal gyrus (L-MTG) (Ch 17: t = 2.28, *p* = 0.024). Other significant brain regions included R-SMG (Ch 23: t = 2.25, *p* = 0.046) and R-PreCG/R opercular part of inferior frontal gyrus (R-IFGoper) (Ch 29: t = 2.25, *p* = 0.040). (See S3 Fig for the grand averaged time courses of the hemodynamic responses for 2 experimental conditions.)

When using HbR changes as an index, permutation *t* tests (S3 Table) showed significant results for both of the Correct and Incorrect conditions versus baseline and compared to one another. All HbR results are summarized in S4 Table.

## Discussion

The current study investigated the neurodevelopmental origins of NAD learning by monitoring infants’ neural responses to NAD processing across 2 fNIRS experiments using an artificial grammar learning paradigm. In Experiment 1, we observed neonates showing significant activation differences between correct and incorrect NAD exemplars after a short Learning phase in left prefrontal regions mainly including DLPFC and FP. Despite no primary activation in either the anterior- or posterior-language areas, the degree of activation in prefrontal regions was negatively associated with the strength of FCs linking prefrontal regions and the posterior-language area during the Learning phase. Additional analyses revealed that the direction of this link may have been driven by the connectivity strength measured during the pre-task resting-state period. In Experiment 2, we found significant activation differences between the Correct and Incorrect NAD conditions in 6- to 7-month-old infants in mainly left-hemispheric brain areas including the left SMG, STG, and IFG with some activations in the right SMG. These findings demonstrate that both neonates and 6- to 7-month-olds can discriminate between correct and incorrect NADs in nonlinguistic contexts after a brief Learning phase. However, the underlying mechanisms for NAD detection are supported by partly different neural processes across age groups. Our results first suggest that even within the first half year of life there is a development toward the use of regions that are part of classical language networks for NAD processing. Second, the FC data demonstrate the involvement of classical language areas (SMG and STG) during NAD learning right after birth and are suggestive of a gradual strengthening of the brain network related to NAD detection as infants are exposed to grammar-like input. Potentially, such a brain network underlying NAD detection could originate from the pre-established FC between prefrontal areas and posterior language areas at birth.

### The prefrontal cortex underlies neonates’ NAD detection

Experiment 1 with neonates shows activation in left prefrontal regions, including DLPFC and FP and IFG. Many prior studies have demonstrated that infants are active learners and, surprisingly, rely more on the prefrontal cortex to support their learning than previously thought [35]. For example, 2 fNIRS studies demonstrated that sleeping 3-month-olds not only detect the violation of the learned rules [39] but also the occurrence of a novel sound [40] through employing the lateral prefrontal cortex. Even neonates’ left IFG seems to be involved in learning speech sounds [5] as well as regularities between syllables, specifically, repetition patterns [41]. Further, sleeping neonates recruited FP and IFG which were connected to the posterior-language areas in response to stimulation of maternal speech [32]. Another recent fMRI study also revealed that the prefrontal cortex may be sufficiently developed during young infancy to support stimulus-driven attention [42]. This evidence from the prefrontal cortex during early infancy is in line with our proposal that neonates’ brains are already sensitive to violations of NADs occurring in novel exemplars of pre-learned tone sequences. Furthermore, the additional activation of left FP in neonates possibly reveals that FP serves as a functional “add-on” at the apex of the hierarchy of lateral prefrontal processes, since FP activations are always concurrently observed together with lateral prefrontal activations [43]. Furthermore, the activation in a small part of IFG (Ch 38) might reveal that the neural underpinnings of processing NADs start involving the anterior-language area.

### Functional networks linked to learning effect in neonates’ prefrontal cortex

Interestingly, neonates did not show activation in the posterior-language areas for NAD detection (i.e., STG and SMG) as observed in 6- to 7-month-olds. However, negative relations between the degree of response in left DLPFC/FP during the Test phase and the strength of FCs linking DLPFC/FP and STG/SMG during the Learning phase indicate a potential neural mechanism including temporo-parietal cortices. Firstly, consistent with previous studies [44,45], neonates showed a large number of FCs either during resting-state or stimulation phases, reflecting active sleep brain states of neonates [46]. However, among those phases, FCs during the Learning phase were stronger than during the Pre-Rest phase, both shown in long-range FCs between anterior-posterior brain regions and between hemispheres. In addition, consistent with findings from 3-month-old infants [44] and adults [47–49], the strength of FCs in the Post-Rest phase was higher or weaker than that in Pre-Rest phases. The FC difference between Pre-Rest and Post-Rest phases indicates that the exposure to sound stimuli during Learning and Test phases shaped resting-state brain networks of neonates, in particular, long-range FCs.

Most interestingly, when exploring the possible mechanisms involving the learning-related brain network, we found that larger NAD detection effects in the prefrontal regions were linked to smaller increments of the left anterior-posterior FCs during the Learning phase compared to the Pre-Rest phase. We interpret this finding in the following way: Neonates equipped with stronger connections as a default state (Pre-Rest) for the learning-related network require less cognitive effort to strengthen the network in learning NADs during the Learning phase, and such facilitated connections may have resulted in enhanced learning performance as reflected in the prefrontal activity measured during the Test phase. In contrast, for those neonates with weaker connections as a default state, a strengthening of the long-range anterior-posterior FCs is needed, as reflected by higher Learning minus Pre-Rest FCs during the Learning phase, but the still inefficient network may have led to weaker detection effects in the Test phase.

The learning-related brain network observed here involved the posterior-language areas including SMG and STG (see Fig 4). This suggests that neonates, despite showing exclusively prefrontal activation for the detection of NAD violations, relied on language-related brain regions during learning. The functional link between those regions may become more stable over time. The involvement of a larger left-hemispheric network for violation detection in 6- to 7-month-olds makes this seem plausible. Beyond language-related functions, the observed functional network could also serve more domain general purposes. Specifically, we speculate that the long-range FCs linking DLPFC and the inferior parietal cortex (e.g., SMG) reflect an early functionality of the already instantiated frontoparietal network, which is engaged in maintaining and manipulating information, since birth [50]. This fronto-parietal network could well play an important role in the service of NAD learning in neonates.

Although neonates detected NAD violations by involving different brain areas compared to 6- to 7-month-olds, the findings on FC analyses revealed that neonates recruit functional brain networks that are probably supported by 2 structurally immature, yet to some degree functional language pathways [31,34]. With auditory exposure and its learning processes via the dorsal pathways, infants may gradually construct the anterior-posterior language network in the first 6 months from birth, which may facilitate future language-related learning. This would eventually result in an enhanced role of SMG and STG for the processing of grammar-like structures. The results of Experiment 2 in 6- to 7-month-olds are consistent with this hypothesis.

### Activation in the posterior-language area for 6- to 7-month-old infants

Here, we present the first evidence of specific brain regions engaging in auditory NAD processing in 6- to 7-month-olds, suggesting that the detection of NAD violations after learning may be subserved chiefly by anterior- and posterior-language areas (IFG, SMG, and STG) in addition to the comparable contralateral regions. This is consistent with findings in 9-month-olds processing syllabic NADs which engaged bilateral temporal regions [14]. Our results extend this finding firstly, by relating functional brain activation to the discrimination of NADs versus NAD violations instead of NAD versus no-NAD conditions [14] and secondly, by probing the brain basis of NAD detection at an even younger age. The activation pattern at 6 to 7 months is, to some extent, already the miniature of a mature adult brain activation in similar paradigms. Previous adult fMRI studies and reviews [51,52] on artificial grammar learning have reported that frontoparietal areas, including left IFGoper, left inferior/superior parietal cortex, are involved in computing the syntactic regularity [28,29,53–55]. In addition, left posterior STG is often reported to be activated in these studies [54,56,57].

Activation of Brodmann’s area 44 (BA 44) has been reported to be associated with the processing of both rather simple as well as more complex linguistic structures [28,29]. As we found activation in IFGoper (i.e., BA 44) and IFGtri (i.e., BA 45) in 6- to 7-month-olds in response to nonlinguistic tone sequences, we suggest that inferior prefrontal regions play a core role in recognizing structural relations in the auditory environment after about half a year of extrauterine language exposure. The large STG response to Incorrect relative to Correct conditions may not only reflect the processing of physical auditory aspects of the stimuli but also computational aspects of processing. A previous ERP study showed that 4-month-olds can discriminate violations of NADs consisting of non-native syllables. These results were discussed in terms of the phonologically based associative learning mechanism mainly rooted in the temporal cortex although the respective experiments did not provide any functional anatomical information [15,34]. Notably, we found activation in the bilateral SMG (one part of the inferior parietal lobe) in Experiment 2. The SMG has previously been associated with phonological working memory processes [58,59] which also played a central role in a word segmentation task by 7- to 10-month-old infants [60]. During the Test phase in the present study, infants may have relied on auditory working memory processes in a similar way. Infants might generate memory-based expectations and their SMG responded upon coming across a violation, namely when the retrieval outcome contradicts those expectations [61]. It is possible that the activation in SMG, as a hub of the frontoparietal attention network, treats incorrect tone sequences presented in the Test phase as salient stimuli due to their unpredictable nature. This could result in infants involuntarily allocating their attention to these salient stimuli [62]. Such an interpretation is consistent with predictive coding theory, which assumes that internal forward models play an important role in many cognitive processes. Both STG and SMG have been suggested as playing a role during the processing of auditory sensorimotor predictions including in language production [63] and comprehension [64]. The activations found for the infants in the present study are consistent with the idea that predictions about upcoming tones in NAD sequences were formed and, in the case of violations, treated as prediction violations.

### NAD learning as an innate ability with ancient phylogenetic roots

Over and above the finding of specific brain regions involved in NAD learning and detection across development, our study demonstrates that this ability may be present in humans starting from birth onwards. It can be found for nonlinguistic stimuli and it does not depend on a mature language network. Previous studies have also demonstrated this ability in both closely and distantly related species including apes, monkeys, rats, and birds [17,65,66]. While the basic ability to learn NADs may thus be present from early on, both ontogenetically and phylogenetically, the extensive use that human language makes of such structures may further shape both the cognitive processes and the neurophysiological substrate that is recruited during NAD processing. Such a view is congruent with human studies showing that the learning of simple NADs and even more complex sequential patterns varies as a function of the developmental status of learners [13,67] and the way the patterns are learned [11]. The present findings may be taken to speculate that language development in the domain of sequential structure piggybacks on infants’ innate abilities to detect adjacent and even nonadjacent auditory patterns which may form an important basis for later syntactic development.

### Limitations

Deactivation was found in both experiments when comparing correct tone triplets with baseline (i.e., standard stimuli). Such deactivation is usually observed in the habituation period (e.g., baseline of mismatch paradigm), when the same or similar types of stimuli are repeated. This is also the case in young infant’s brains [40,68], as for example 3- to 4-month-old infants showed a decreased pattern of hemoglobin changes in response to repetitive sounds [40]. We interpret the deactivation in response to the correct triplets as a reflection of habituation. Although the correct stimuli were not acoustically the same as the standard stimuli presented in the baseline with respect to pitch, infants are very likely to perceive those correct triplets and standard triplets as the same in the context of the NAD rule, therefore, resulting in habituated activation. As both experiments further yielded activation increases for novel incorrect stimuli compared to novel correct ones, we must conclude that the underlying NAD rule led to a further differentiation of the 2 activation patterns. Although a difference between novel incorrect versus baseline stimuli in newborns was not significant, the critical results supporting discrimination ability are the extent of differences between 2 activation patterns. Specifically, quite opposite activation directions (i.e., deactivation to the novel correct and activation to the novel incorrect) were observed for both groups and such difference were significant. Thus, we maintain the conclusion that both newborns and older infants extracted the NAD rule while acknowledging that the sensitivity in detecting the rule violation may be slightly weaker in newborns.

Note that we used nonlinguistic tones in our experiments in order to study processes that are especially important for language processing. The underlying aim was to minimize the effect of prenatal language experience and processing load and to ensure comparability with animal research. While differences between tonal and speech stimuli have been reported during NAD learning in later infancy and early childhood studies [13], within the first half year of life rather report evidence for NAD learning in both domains [12,15]. Furthermore, a review of developmental hemispheric lateralization in language acquisition [69] posits a model which assumes left-hemispheric learning biases for both speech and non-speech sounds. Thus, we assume comparability of basic involved mechanisms of linguistic and nonlinguistic NAD learning in the first year of life while we acknowledge the need to further corroborate this in direct comparisons across modalities.

Another limitation of this study is that the infants’ states differed between Experiments 1 and 2. Measurements with neonates were performed during the active sleep state, but measurements with 6- to 7-month-old infants were performed during the awake state. Taga and colleagues [46] systematically examined the differences in brain activation and task-based FC with infants aged 2 to 10 months. They found that auditory stimuli produced a global activation for the sleep state but a focal activation for the awake state. Therefore, in the case of newborns, more focal activations in prefrontal regions would be observed for awake newborns, unlike our current results. However, in comparing the 2 age groups, activation foci were, in principle, clearly different between our 2 experiments, namely the left prefrontal area for sleeping neonates and the left temporo-parietal area for the awake 6- to 7-month-olds. These data imply sleeping state does not act as an obvious confound and hence should not impact the interpretation of our findings. Furthermore, as mentioned before, even within newborn results, the sleeping state may not change the results much, considering the results of previous studies [46]. However, the functional reorganization of brain regions supporting NAD learning across the first half year of life still needs to consider the state difference of infants in our 2 experiments.

To avoid higher attrition when collecting data from 6- to 7-month-old infants using probes with 44 channels over a long measurement duration, in line with previous meta-analysis studies [70,71], we decided to monitor the hemodynamic responses only during the Test phase. With advances in fNIRS hardware development, we believe that lighter headgear will enable the recording of approximately 20-min hemodynamic responses in future studies while obtaining relatively clean data. This will provide us with an opportunity to better investigate the developmental changes in language acquisition.

To conclude, our study uses fNIRS to shed new light on the neurodevelopmental origins of a crucial building block of syntax—the capacity to process NADs. Specifically, we provide the first evidence that neonates are capable of extracting NADs from auditory sequences. Yet, during NAD detection neonates relied on different brain regions compared to 6- to 7-month-old infants on the same task. This suggests a rapid development of brain networks relevant to grammar-like processing within the first 6 months of life. Specifically, 6- to 7-month-old infants already use the same brain regions (IFG and the posterior-language area) as adults to track NADs, while neonates recruited solely prefrontal regions. Neonates’ FCs during learning additionally revealed a learning-related brain network including posterior language-related areas that were found for NAD detection in 6- to 7-month-olds. These findings add to the growing evidence that the prefrontal cortex in concert with functionally connected posterior areas supports early cognitive development and indicates that the infant brain has already been equipped with an extraordinary learning ability as a precursor for later grammar acquisition.

## Materials and methods

### Participants

For Experiment 1, 21 full-term healthy neonates (11 female, mean age = 3.5 days, SD = 0.9, range: 1 to 5 days) contributed data to the final analyses. An additional 2 neonates were tested but were excluded due to fussiness/awake during the experiment. The number of neonates included in the FC analysis with valid FC data was 18, 17, 15 for the Pre-Rest phase, the Learning phase, and the Post-Rest phase, respectively. All neonates had normal hearing which was assessed using auditory brainstem responses or other clinical tests. Mean birth weight for neonates was 2,822 ± 358 g (range: 2,078 to 3,527 g). For Experiment 2, 19 healthy 6- to 7-month-old full-term infants (6 female, mean age = 201.2 days, SD = 9.8: range, 185 to 223 days) were included in the final analysis. Additional 22 infants were excluded from the analysis because of either rejection of wearing the fNIRS probe cap (*n* = 1), cessation of the experiment due to frequent fussiness (*n* = 12), or insufficient blocks caused by motion artifacts (*n* = 9). Written informed consent was obtained from parents before participation. The current fNIRS study was designed and performed according to the principles of the Declaration of Helsinki. Experiments 1 and 2 were approved by the ethics committee of Keio University Hospital (No. 20090189) and the ethics committee of Keio University, Faculty of Letters (Reference Number: 18009), respectively.

### Stimuli

The stimuli were identical to the materials used in a previous comparative study on NAD learning [17]. Each of artificial grammar sequences was composed of 3 elements from 6 computer-generated acoustic categories (A, B, C, D, X1, and X2) of frequency-modulated sine tones. As the duration of each element was 1.5 s and the time interval between 2 adjacent elements was 0.25 s, each artificial grammar sequence lasted 5 s. Each category was characterized by its specific pitch contour (e.g., A: rising; B: slow wave; C: fast wave; D: falling; X1: big spike; X2: small spike; Fig 1A). The acoustic categories A, B, C, and D were composed of 10 pitch-shifted variants (S5 Table). Each category variants from 1 to 5 started from 500 Hz to 700 Hz, with a separation of 50 Hz between 2 adjacent variants, and variants from 6 to 10 ranging from 900 Hz to 1,100 Hz. A 200-Hz gap between variants 5 and 6 of each category was manipulated to increase the perceptual difference between ranges 1 to 5 and 6 to 10. The acoustic categories X1 and X2 consisted of 16 pitch-shifted variants (S6 Table). Each category variants from 1 to 8 started from 500 Hz to 710 Hz, with a separation of 30 Hz between 2 adjacent variants, and variants from 9 to 16 ranging from 900 Hz to 1,100 Hz. A 190-Hz gap between variants 8 and 9 of each category was inserted to increase the perceptual difference between ranges 1 to 8 and 9 to 16. All elements were generated using Praat [72]. We created 2 sets of paired NAD grammars, i.e., Grammars 1 and 2. For Grammar 1, A elements were always followed by B elements (Grammar 1a), and C elements by D elements (Grammar 1b), whereas the middle intervening X elements varied freely. Conversely, the roles of B and D elements were reversed for Grammar 2, with D dependent on A (Grammar 2a) and B dependent on C (Grammar 2b). To control for the possibility that certain sound pairings might be relatively easier to learn, we created 4 stimulus lists, each of which consisted of the standard triplets, the correct triplets, and the incorrect triplets (S7 Table). The standard triplets and the correct triplets in one of 4 stimulus lists were used as one of 2 artificial grammars (e.g., Grammar 1) while the incorrect triplets as another one of 2 artificial grammars (e.g., Grammar 2).

### Procedure

Experiment 1 was conducted in a testing room at the hospital and consisted of 4 phases, that is the Pre-Rest phase, the Learning phase, the Test phase, and the Post-Rest phase (Fig 1B and 1C). All neonates were tested while they were in natural sleep. Given that more than half of the sleeping period in neonates is spent in an active sleep state [73], we assumed that neonates in Experiment 1 were in an active sleep state during the experiment, based on visual inspection and the general time schedule of their daily care routine. In the Learning phase, neonates were presented with a total of 60 standard triplets (e.g., Grammar 1: 30 triplets for Grammar 1a and 30 triplets for Grammar 1b) drawn from one of the 4 stimulus lists (S7 Table) via 2 speakers positioned 45 cm from their heads while they were sleeping. Each triplet lasted 5 s and the time interval between 2 triplets was 1.5 s. Thus, 60 standard triplets resulted in the Learning phase lasting approximately 6 min. We used a block design in the Test phase such that the 2 types of target trials, Correct and Incorrect, were spaced by baseline trials varying duration (11.5 s, 18 s, and 24.5 s) to avoid synchronization between stimuli occurrences and spontaneous oscillations. The 2 target trial types were presented in a pseudorandom order, which was constrained to prevent more than 2 consecutive blocks of the same condition. The order of the trials was counterbalanced across neonates. A Correct trial was composed of 2 correct triplets, while an Incorrect trial included 2 incorrect triplets. Each triplet was 5 s and the time interval between 2 triplets was 1.5 s, meaning each target trial lasted 11.5 s in total. For one given neonate, the stimuli for the Correct and Incorrect trials in the Test phase were selected from the same stimulus list as that presented in the Learning phase. Assignment to 4 stimulus lists was counterbalanced across neonates as much as possible. The Test phase lasted for approximately 7.5 min since almost all neonates completed 10 trials for each experimental condition. Besides, before the Learning phase and after the Test phase, 2 resting-state phases were incorporated to examine FC when neonates received no explicit tasks.

The procedure of Experiment 2 was slightly different from Experiment 1 in that the former consisted of only 2 phases, that is the Learning phase and the Test phase (Fig 1C). During the Learning and Test phases in Experiment 2, the same stimuli as Experiment 1 were presented to 6- to 7-month-old infants in a sound-attenuated room. The infant sat on a mother’s lap and 1 loudspeaker was located approximately 1.5 m from the infant. An experimenter entertained the infant with silent toys, or a silent cartoon movie played on an iPad to avoid their fussiness. In the case of the silent toys (plastic blocks and small stuffed toys), the experimenter moved the toys within the infant’s visual field to avoid a big motion artifact. The mother was instructed not to move and speak to her infant if it was not necessary. Additionally, the mother and the experimenter listened to music through headsets to prevent any potential influence on the infant’s behavior. After the Learning phase consisting of 60 standard triplets, 2 experimenters placed fNIRS probe pads onto the infant’s head. After the intensity level of all the channels had been checked, the Test phase immediately began. Here, we monitored the hemodynamic responses of infants exclusively during the Test phase to avoid the high attrition rate for infants at 6 months due to the long scan time. We ended the experiment after 10 complete trials for each experimental condition, or when the infant became too fussy as judged by the experimenter.

To prevent the discomfort of participants, we used particular types of probe pads, which are made of silicon with small emission and detection sensors inside them and specially made for young infants in both experiments. In the case of newborns, their saturation levels were monitored throughout the entire experiment. All the procedures were video recorded for use in data analysis (i.e., artifact check).

### fNIRS measurements

The fNIRS measurements in Experiment 1 were conducted using a multichannel NIRS instrument (ETG-4000, Hitachi Medical Co., Japan) with 2 wavelengths (approximately 695 and 830 nm) and a sampling frequency of 10 Hz to record the brain activity of neonates while lying in a supine position. A 3 × 5 and two 3 × 3 rectangular silicon probe pads with the source-detector separation of 20 mm were used to cover the frontal, temporal, and parietal regions, yielding a total of 46 channels (S1A and S1C Fig). The 3 × 3 probe pads for the bilateral temporal regions were placed according to T3 and T4 in the 10–20 system so that the midpoint between Ch 11 and Ch 12 (or between Ch 23 and Ch 24) corresponded to T3 (or T4). The lowest probe row was nearly aligned with the horizontal reference curve (F7-T3-T5 or F8-T4-T6). The lowest probe row of the 3 × 5 probe pad was placed along with the horizontal reference curve (F7-Fp1-Fpz-Fp2-F8) for the frontal region so that the midpoint between Ch 26 and Ch 27 was at Fpz. The middle column was aligned along the sagittal reference curve. For neonates, the mean head circumference, mean distance from the left to right ear via the top of the head, and mean distance between the nasion and inion via the top of the head were 33.6 cm (SD = 1.3), 22.3 cm (SD = 1.2), and 22.3 cm (SD = 1.0), respectively. Because the arrangement of probe pads in this experiment is completely the same as a recent study [32], we also used a modified version of the virtual registration method estimated the most probable Montreal Neurological Institute coordinate values. And then the AAL atlas [74] and Broadmann atlas were used to determine the brain regions for each channel covered by 2 bilateral probe pads and a frontal probe pad, respectively. The detailed descriptions regarding the estimation of brain regions in the current probe arrangement could be found in Uchida-Ota and colleagues [32].

For Experiment 2, we used a multichannel NIRS instrument (ETG-7000, Hitachi Medical Co., Japan) with 2 wavelengths (approximately 780 and 830 nm) and a sampling frequency of 10 Hz. We used two 3 × 5 rectangular silicon probe pads specifically designed for 6- to 7-month-old infants, each of which incorporated 8 sources and 7 detectors separated by 20 mm, yielding a total of 22 channels (S1B Fig). The 3 × 5 probe pads were placed according to T3 and T4 in the 10–20 system so that the midpoint between Ch 21 and Ch 22 (or between Ch 41 and Ch 42) corresponded to T3 (or T4). The lowest lines of probe pads were positioned parallel to the T3-Fp1-Fp2-T4 line. For the 6- to 7-month-old infants, the mean head circumference, mean distance from the left to right ear via the top of the head, and mean distance between the nasion and inion via the top of the head were 43.3 cm (SD = 1.3), 29.7 cm (SD = 1.9), and 28.6 cm (SD = 1.1), respectively. We utilized the virtual registration method [75] and the AAL atlas [74] to estimate brain regions underlying each channel by adjusting the source-detector separation to consider the difference between adult and infant head size. Despite different probe pads for Experiments 1 and 2, brain activities from frontal, temporal, and parietal regions were recorded according to the 10–20 system.

### Data and statistical analysis

For Experiment 1: neonates, fNIRS data preprocessing was carried out on the MATLAB-based software Platform for Optical Topography Analysis Tools (POTATo, Hitachi, Research and Development) [76]. Based on the modified Beer–Lambert law [77], the optical density data were transformed into the product of HbO or HbR concentration change and optical path length, which was defined as ΔHbO or ΔHbR (in mM·mm), respectively. Trials treated as contaminated by motion artifacts were excluded, according to the criteria for rapid signal change in the sums of ΔHbO and ΔHbR (>0.15 mM·mm changes within 2 consecutive data points). Trials with a saturated light intensity value (cf. more than 5 mM·mm) were also removed. For each neonate, data containing a minimum of 2 valid trials per condition and at least half of valid channels was included in the final data set. The time course of ΔHbO and ΔHbR then band-pass filtered using a third-order Butterworth filter between 0.02 and 0.5 Hz to eliminate slow drift and cardiac pulsation. Subsequently, the continuous data were segmented into blocks consisting of a 5-s baseline, 11.5-s experimental stimuli (Correct or Incorrect condition), and 11.5-s poststimulus baseline. For these 28-s blocks, a baseline was linearly fitted between the first and last 5 s of each block. Lastly, we averaged all valid blocks for each channel per neonate. The average valid blocks across all neonates and channels were 6.9 (SD = 0.9) for Incorrect and 7.4 (SD = 1.5) Correct conditions, respectively. Group average waveforms from 2 experimental conditions (Correct and Incorrect conditions) were constructed over all neonates and channels to show the overall hemodynamic response shape so as to choose a time window of interest. We conducted the statistical analysis using the mean value of ΔHbO/ΔHbR within the time window of 7.5 s to 12.5 s from stimulus onset in this experiment. For each channel, statistical comparisons (paired sample permutation *t* tests) of average ΔHbO/ΔHbR during the specified time windows were performed for the (i) Correct or Incorrect condition compared to the baseline condition; and the (ii) direct comparison of the Correct and Incorrect conditions; 50,000 random permutations were implemented to estimate the distribution of the null hypothesis for an alpha level of 0.05. Because no significant results from ΔHbR were found, we primarily reported findings from ΔHbO, which also suggested that ΔHbO is a more sensitive index, as indicated by previous studies [60,78].

In addition, we also performed the FC analyses. The preprocessing of continuous fNIRS data during the Pre-Rest, Learning, and Post-Rest phases was performed using the Homer2 package [79]. First, we converted the raw intensity data into changes in optical density data (ΔOD). Then, the wavelet motion correction algorithm was applied to the time course of ΔOD within each channel independently using the infant-specific tuning parameter iqr = 0.5 [80,81] to detect and remove motion artifacts. We thereafter applied basic trial rejection to identify and remove residual motion artifacts by the Homer2 function hmrMotionArtifactByChannel with the infant-specific tuning parameter thresholds: tMotion = 1 s, tMask = 1 s, stdThresh = 15, and ampThresh = 0.4 [81]. The resulting data were band-pass filtered in the frequency range of 0.01 to 0.1 Hz to reduce the effect of high-frequency noise and baseline drift, and to obtain the low-frequency hemodynamic signals that emanated from spontaneous neural activity [46,82–84], followed by conversion of ΔOD data to change in HbO and HbR using modified Beer–Lambert law with a differential pathlength factor, DPF = 5 [85]. If data included one “bad” channel due to motion artifacts or a saturated light within the continuous 90 s, it was excluded. Thus, the number of participants with valid FC data was 18, 17, 15 for the Pre-Rest, Learning, and Post-Rest phases, respectively. Finally, we extracted more than 90-s data (including more than 900 sample points) from continuous time courses of 46 channels for each neonate to perform further FC analyses. The average duration of all valid time course data was 183.3 s (SD = 58.0, range: 90.0 to 240.0), 291.2 s (SD = 98.7, range: 100.0 to 390.0), and 202.5 s (SD = 34.2, range: 150.0 to 230.0) for the Pre-Rest, Learning, and Post-Rest phases, respectively. The duration of Learning phase was significantly longer than that of Pre-Rest phase (t(14) = −3.78, *p* = 0.002), but no significant difference between Pre-Rest and Post-Rest phases was observed (t(13) = −0.34, *p* = 0.742).

In order to obtain significant FCs during the Pre-Rest, Learning, and Post-Rest phases, the first step of our analysis was to calculate the Pearson correlation coefficients (r) between the whole time course of HbO change from (46 × 45)/2 = 1,035 pairs of channels for each neonate. We then converted r values to Z-values via Fisher’s r-to-z transformation to improve normality. One-sample *t* tests were applied to find the significant FCs for each phase against a zero baseline. The cut-off levels of significance were set at 5 thresholds (*p* < 0.05, *p* < 5e-6, *p* < 5e-7, *p* < 5e-8, and *p* < 5e-9), and multiple comparisons among 1,035 FCs were corrected using the Bonferroni method. The second step of our analysis was to perform paired *t* tests to determine FCs with significant changes relative to Pre-Rest by comparing FCs between Pre-Rest and Learning phases (Learning versus Pre-Rest), as well as FCs between Pre-Rest and Post-Rest phases (Post-Rest versus Pre-Rest). And when comparing FC differences for the contrasts of Learning versus Pre-Rest and Post-Rest versus Pre-Rest, the number of valid neonates was 15 and 14, respectively. The third step of our analysis was to examine whether there were significant associations between FCs with significant changes during/after NAD rule learning and the prefrontal activation engaged in NAD rule detection. We firstly chose 6 prefrontal channels (Ch 30, Ch 34, Ch 35, Ch 38, Ch 39, and Ch 43) that were significantly activated during the Test phase as seed channels, and then we calculated the Pearson correlation coefficients between the strength of FCs with significant changes (i.e., Learning minus Pre-Rest; Post-Rest minus Pre-Rest) and the degree of response in 6 prefrontal seed channels. For this analysis, only FCs which included any one of those 6 seed channels (i.e., seed-based FCs) were examined, because we aimed to find FCs involved in NAD learning (Fig 4A). The fourth step of our analysis was to examine whether these FCs formed a local brain network in response to NAD learning together with other FCs connecting any 2 non-seed channels (Ch 2, Ch 9, Ch 17, Ch 19, and Ch 22). Thus, we searched for FCs whose strength was significantly correlated with activation in 6 seed channels among all FCs with significant changes and simultaneously including non-seed channels (Ch 2, Ch 9, Ch 17, Ch 19, and Ch 22). Given the strength of FCs within the local brain network underlying the NAD learning was negatively related to the prefrontal activation found for NAD violation detection, an additional analysis was performed to further explore the mechanism of the local brain network. Specifically, we analyzed the relationship between FC strength during the Pre-Rest phase (Pre-Rest minus 0) and that during the Learning phase (Learning minus 0), as well as the relationship between FC strength during the Pre-Rest phase (Pre-Rest minus 0) and FC strength changes from the Pre-Rest to Learning phases (Learning minus Pre-Rest). Likewise, among 19 FCs with significant changes from the Pre-Rest phase to Post-Rest phase, although we only found 1 FC which was negatively associated with the activity in the prefrontal channel, we also examined the relationship between FC strength during the Pre-Rest phase (Pre-Rest minus 0) and FC strength changes from the Pre-Rest to Post-Rest phases (Post-Rest minus Pre-Rest).

For the analysis of the task data collected during the Test phase in Experiment 2 (6- to 7-month-old infants), the data preprocessing and statistical analysis were identical to Experiment 1 (neonates), except that the mean value of ΔHbO/ΔHbR within the time window of 10.5 s to 15.5 s from stimulus onset was used for statistical comparisons according to overall hemodynamic response shape over all infants and channels. The average valid blocks across all infants and channels were 7.0 (SD = 2.1) for Incorrect and 7.1 (SD = 1.9) Correct conditions, respectively. As all fNIRS data preprocessing was carried out on MATLAB-based toolboxes, no custom scripts are available. The scripts for analyzing the task-based activation and FC can be found at https://osf.io/84yu9/ or https://doi.org/10.5281/zenodo.13636036.

## Supporting information

S1 Fig Probe arrangement for fNIRS measurements. (A) The 3D anatomical position for each channel in Experiment 1: neonates. Ch 31 and Ch 40 locates along the longitudinal fissure of the brain. (B) The 3D anatomical position for each channel in Experiment 2: 6- to 7-month-old infants. (C) The 2D graph of channel positions for displaying the FC results in Experiment 1: neonates. Each channel is indicated by a number. Red dots indicate the reference points of the 10–20 system.
(TIF)

S2 Fig **The grand averaged time courses of the hemodynamic responses derived from ΔHbO and ΔHbR for different experimental conditions** (i.e., Correct and Incorrect conditions) for Experiment 1: neonates. Channels are arranged according to the S1C Fig. The data underlying this figure can be found at https://osf.io/84yu9/.
(TIF)

S3 Fig **The grand averaged time courses of the hemodynamic responses derived from ΔHbO and ΔHbR for different experimental conditions** (i.e., Correct and Incorrect conditions) for Experiment 2: 6- to 7-month-old infants. Channels are arranged according to the S1B Fig. The data underlying this figure can be found at https://osf.io/84yu9/.
(TIF)

S4 Fig The FC patterns in Experiment 1. The significantly increased FCs for 3 phases against a respective zero baseline. The data underlying this figure can be found at https://osf.io/84yu9/.
(TIF)

S5 Fig Changes in FC strength from the Pre-Rest phase (Pre-Rest minus 0) to the Learning phase (Learning minus 0) for 4 representative connections. The colored lines and circles represent individual data from 15 neonates. The data underlying this figure can be found at https://osf.io/84yu9/.
(TIF)

S6 Fig (A) A negative correlation between prefrontal activation during the Test phase and strength of one FC with significant changes from the Pre-Rest phase to Post-Rest phase. Left: 2D map of FC, where 6 prefrontal seed channels are indicated with 6 different colors. Right: the scatter plot of such a negative correlation. The blue dots represent data from 13 neonates. (B) A negative correlation between FC strength during the Pre-Rest phase and FC strength changes from the Pre-Rest phase to Post-Rest phase (Post-Rest minus Pre-Rest). The data underlying this figure can be found at https://osf.io/84yu9/.
(TIF)

S1 Table Statistical results of significant paired permutation *t* tests under each contrast: Correct vs. baseline, Incorrect vs. baseline, and Incorrect vs. Correct conditions for ΔHbO in Experiment 1. (DOCX)

S2 Table Statistical results of significant paired permutation *t* tests under each contrast: Correct vs. baseline, Incorrect vs. baseline, and Incorrect vs. Correct conditions for ΔHbR in Experiment 1. (DOCX)

S3 Table Statistical results of significant paired permutation *t* tests under each contrast: Correct vs. baseline, Incorrect vs. baseline, and Incorrect vs. Correct conditions for ΔHbO in Experiment 2. (DOCX)

S4 Table Statistical results of significant paired permutation *t* tests under each contrast: Correct vs. baseline, Incorrect vs. baseline, and Incorrect vs. Correct conditions for ΔHbR in Experiment 2. (DOCX)

S5 Table Base frequency of pitch-shifted variants for acoustic categories A, B, C, and D. (DOCX)

S6 Table Base frequency of pitch-shifted variants for acoustic categories X1 and X2. (DOCX)

S7 Table Stimulus lists used in Experiments 1 and 2. (DOCX)

## Abbreviations

Ch: channel
DLPFC: dorsolateral prefrontal cortex
DPF: differential pathlength factor
ERP: event-related potential
FC: functional connectivity
fMRI: functional magnetic resonance imaging
fNIRS: functional near-infrared spectroscopy
FP: frontopolar
HbO: oxygenated hemoglobin
HbR: deoxygenated hemoglobin
IFG: inferior frontal gyrus
IFGtri: inferior frontal gyrus pars triangularis
NAD: nonadjacent dependency
preCG: pre-central gyrus
SMG: supramarginal gyrus
STG: superior temporal gyrus
