# Supplementary figures and images for: Functional reorganization of brain regions supporting artificial grammar learning across the first half year of life
Source: PLoS Biol. 2024 Oct 22;22(10):e3002610. doi: 10.1371/journal.pbio.3002610 (PMC11495551; doi:10.1371/journal.pbio.3002610)

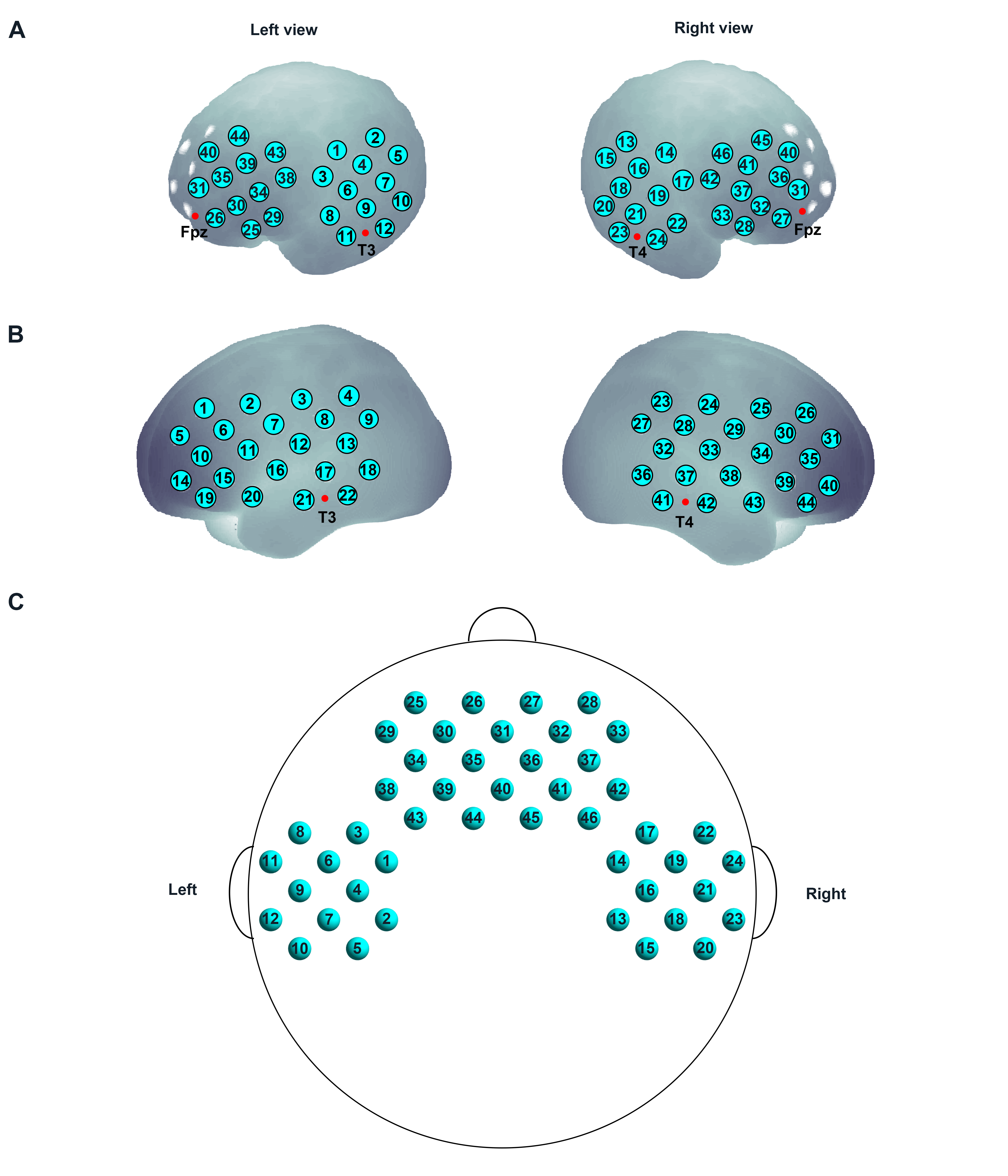

Supplement: S1 Fig — (A) The 3D anatomical position for each channel in Experiment 1: neonates. Ch 31 and Ch 40 locates along the longitudinal fissure of the brain. (B) The 3D anatomical position for each channel in Experiment 2: 6- to 7-month-old infants. (C) The 2D graph of channel positions for displaying the FC results in Experiment 1: neonates. Each channel is indicated by a number. Red dots indicate the reference points of the 10–20 system. (TIF) [file pbio.3002610.s001.tif]

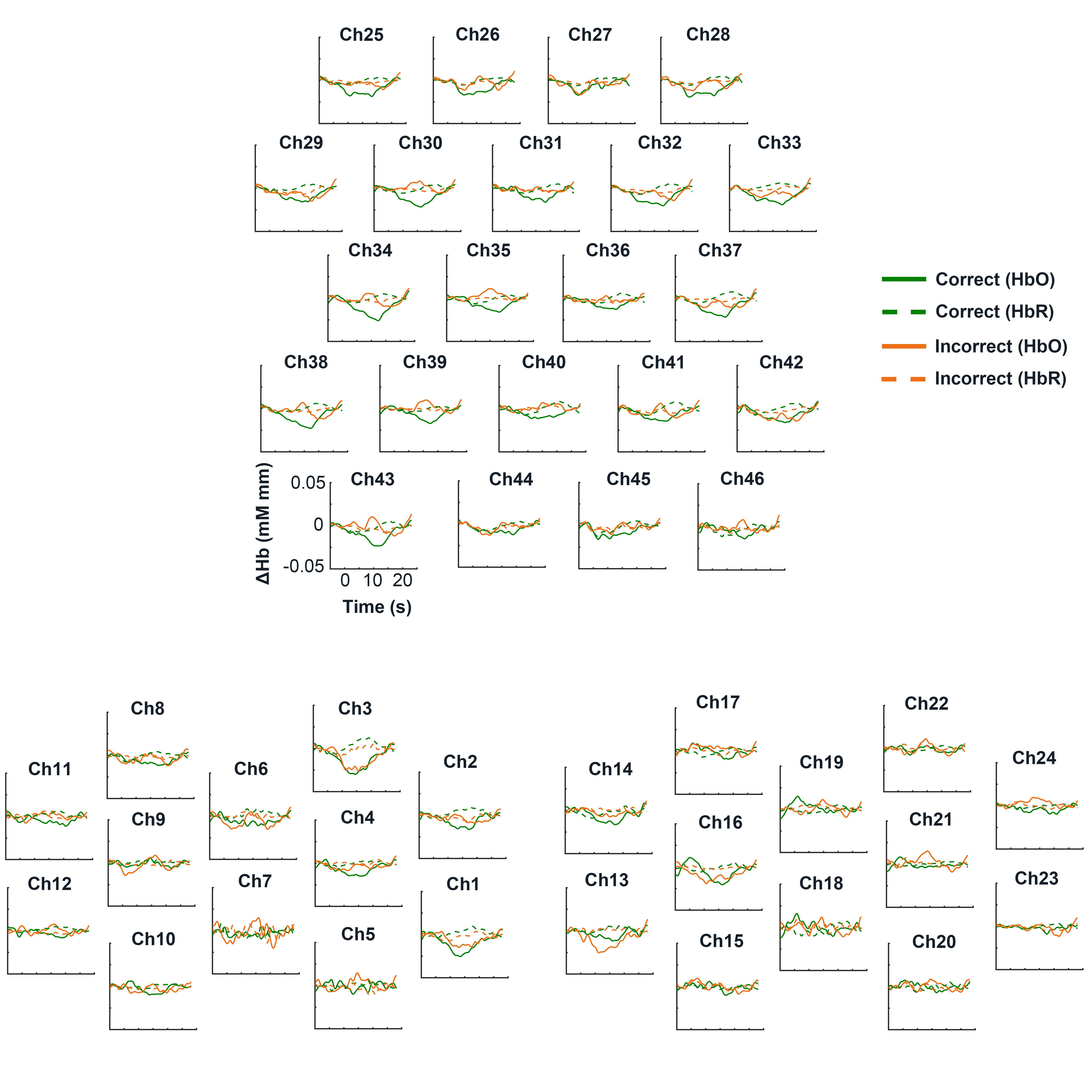

Supplement: S2 Fig — The grand averaged time courses of the hemodynamic responses derived from ΔHbO and ΔHbR for different experimental conditions (i.e., Correct and Incorrect conditions) for Experiment 1: neonates. Channels are arranged according to the S1C Fig. The data underlying this figure can be found at https://osf.io/84yu9/. (TIF) [file pbio.3002610.s002.tif]

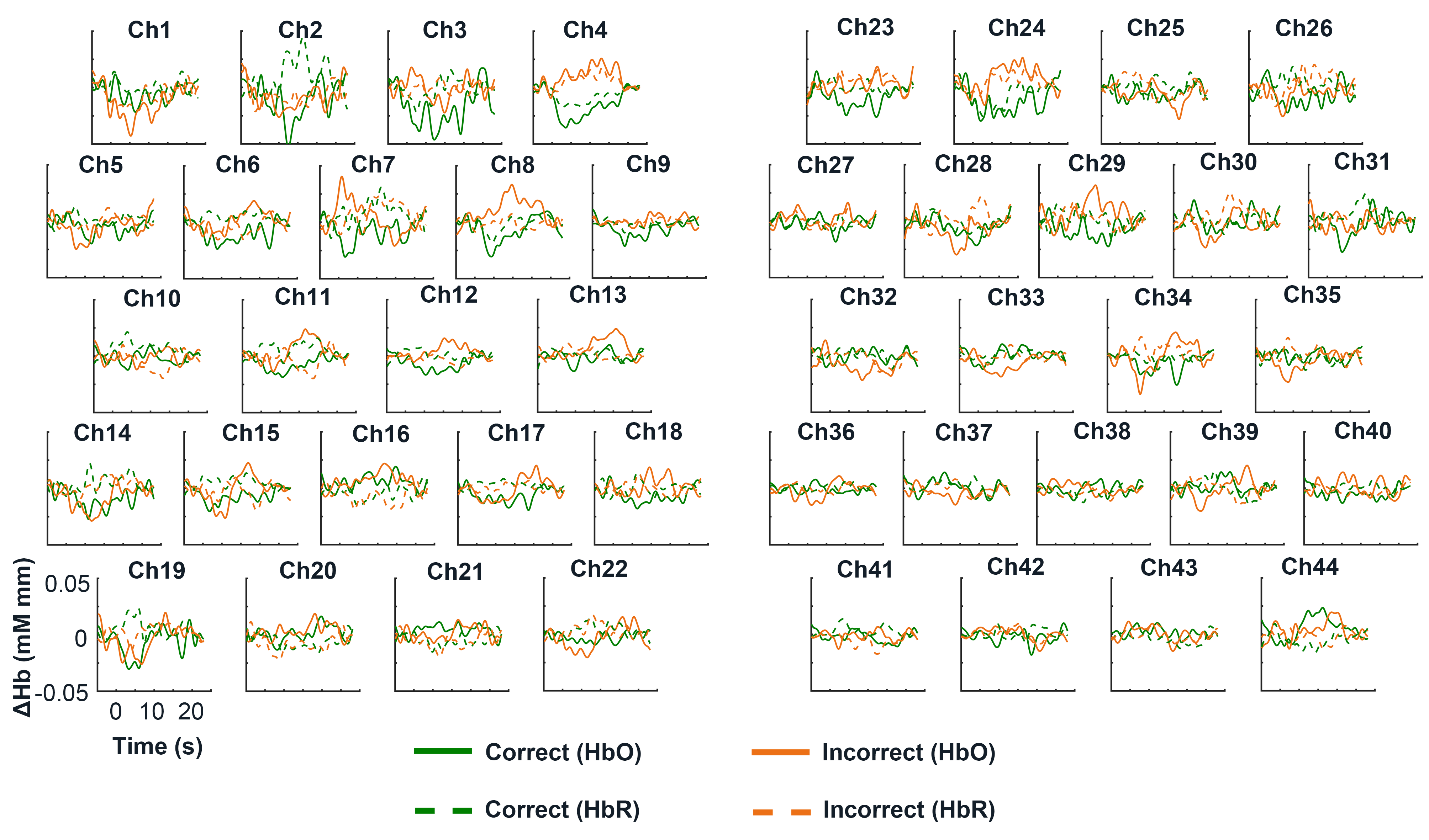

Supplement: S3 Fig — The grand averaged time courses of the hemodynamic responses derived from ΔHbO and ΔHbR for different experimental conditions (i.e., Correct and Incorrect conditions) for Experiment 2: 6- to 7-month-old infants. Channels are arranged according to the S1B Fig. The data underlying this figure can be found at https://osf.io/84yu9/. (TIF) [file pbio.3002610.s003.tif]

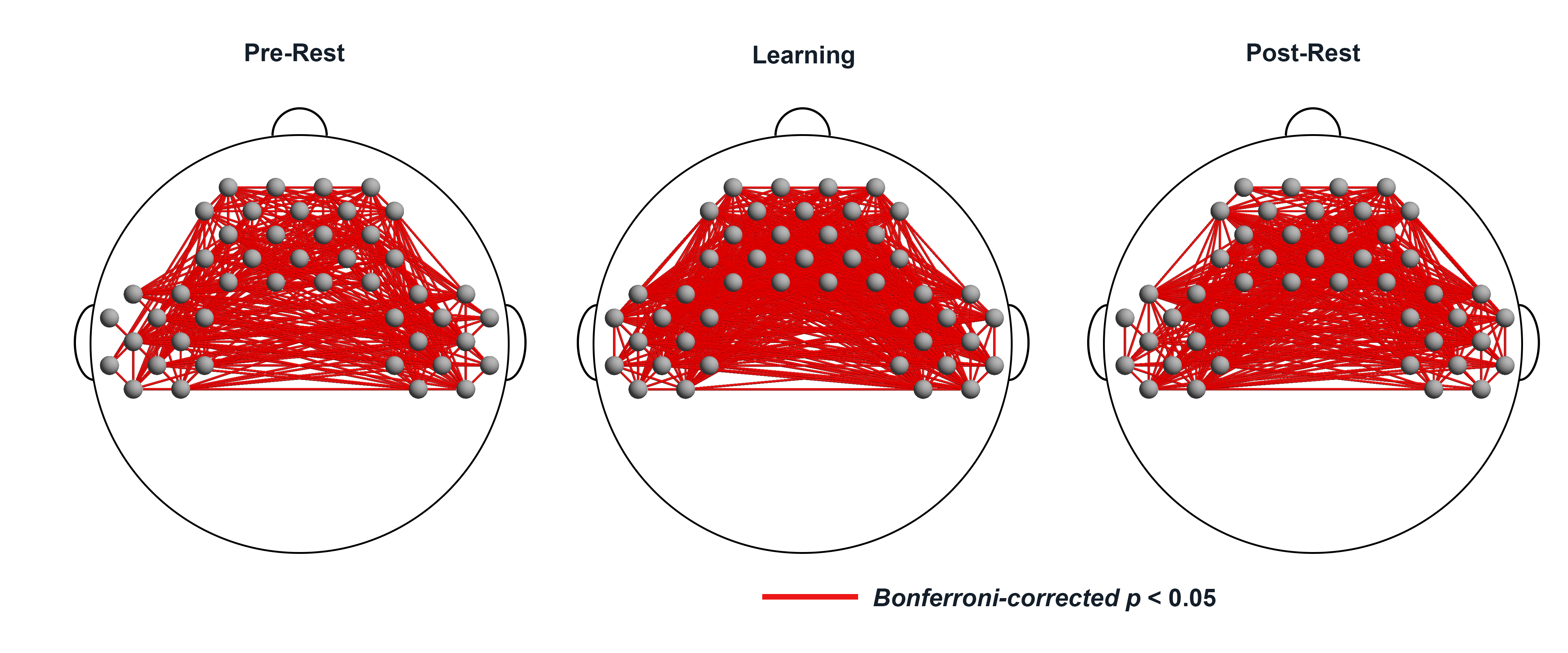

Supplement: S4 Fig — The significantly increased FCs for 3 phases against a respective zero baseline. The data underlying this figure can be found at https://osf.io/84yu9/. (TIF) [file pbio.3002610.s004.tif]

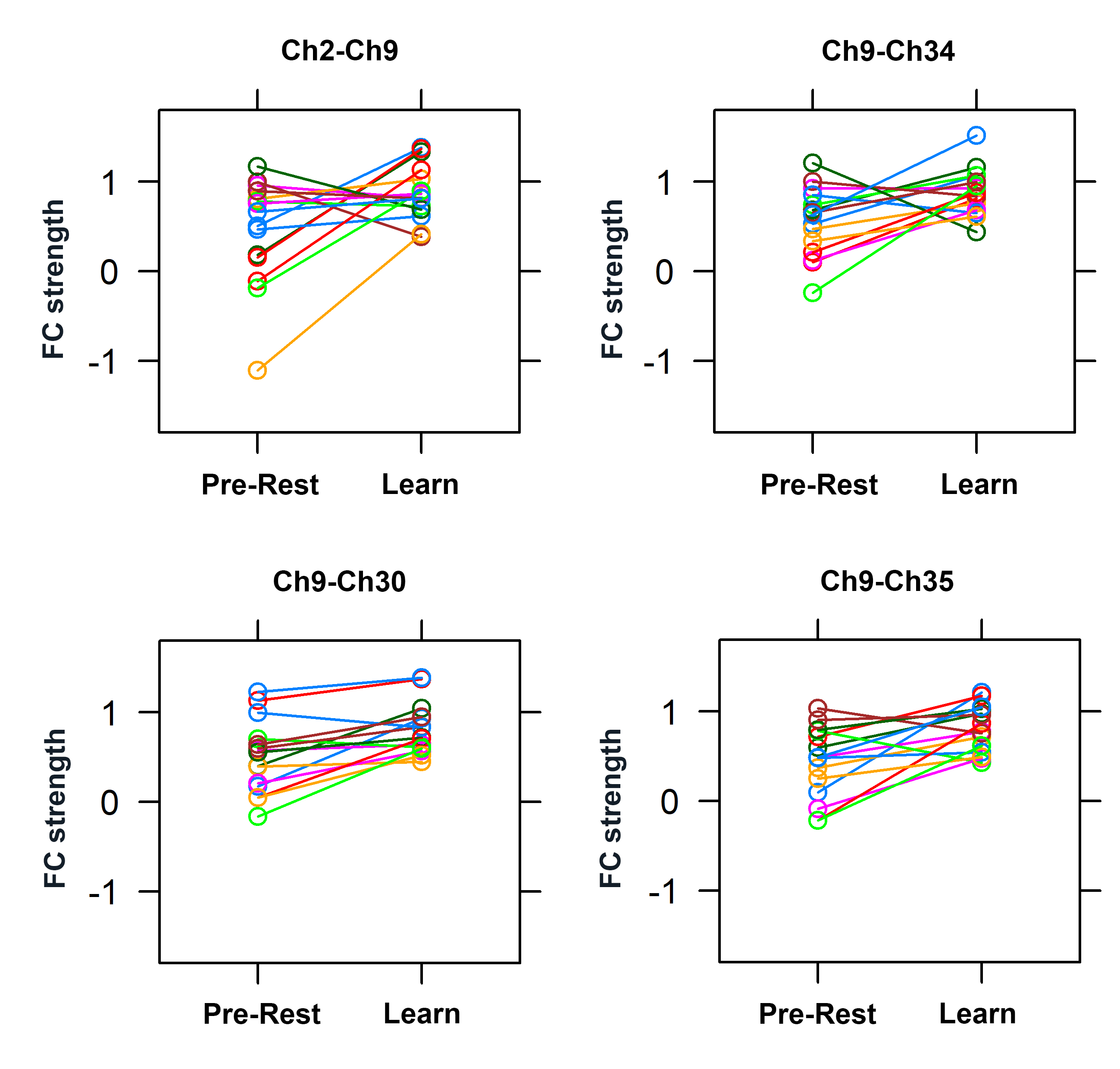

Supplement: S5 Fig — The colored lines and circles represent individual data from 15 neonates. The data underlying this figure can be found at https://osf.io/84yu9/. (TIF) [file pbio.3002610.s005.tif]

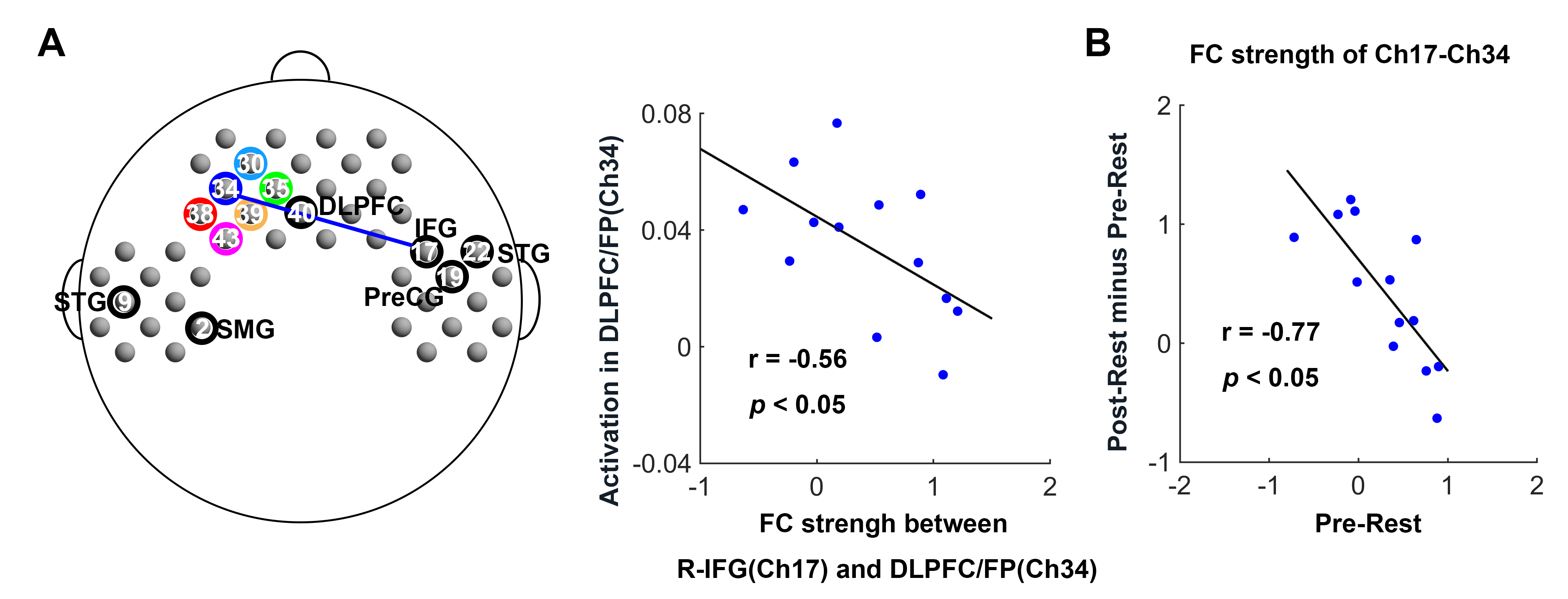

Supplement: S6 Fig — (A) A negative correlation between prefrontal activation during the Test phase and strength of one FC with significant changes from the Pre-Rest phase to Post-Rest phase. Left: 2D map of FC, where 6 prefrontal seed channels are indicated with 6 different colors. Right: the scatter plot of such a negative correlation. The blue dots represent data from 13 neonates. (B) A negative correlation between FC strength during the Pre-Rest phase and FC strength changes from the Pre-Rest phase to Post-Rest phase (Post-Rest minus Pre-Rest). The data underlying this figure can be found at https://osf.io/84yu9/. (TIF) [file pbio.3002610.s006.tif]
