# Supplementary material for: Functional reorganization of brain regions supporting artificial grammar learning across the first half year of life
Source: PLoS Biol. 2024 Oct 22;22(10):e3002610. doi: 10.1371/journal.pbio.3002610 (PMC11495551; doi:10.1371/journal.pbio.3002610)
Supplement: S1 Table — (DOCX) [file pbio.3002610.s007.docx]

**S1 Table.** Statistical results of significant paired permutation t-tests under each contrast: Correct vs baseline, Incorrect vs baseline, and Incorrect vs Correct conditions for ΔHbO in Experiment 1.

| Ch | *t* | *p* | Brain region |
| --- | --- | --- | --- |
| **Correct > baseline** | | |  |
| 3 | -3.13 | 0.005 | L-IFGtri/L-IFGoper |
| 25 | -2.89 | 0.009 | L-FP/L-VFR |
| 26 | -2.69 | 0.015 | L-FP |
| 28 | -2.46 | 0.023 | R-VFR/R-FP |
| 29 | -2.22 | 0.039 | L-DLPFC/L-FP |
| 30 | -3.61 | 0.002 | L-FP |
| 31 | -3.26 | 0.005 | FP |
| 32 | -3.39 | 0.003 | R-FP |
| 33 | -2.84 | 0.011 | R-DLPFC/R-FP |
| 34 | -4.21 | 0.001 | L-DLPFC/L-FP |
| 35 | -2.97 | 0.009 | L-FP |
| 36 | -2.48 | 0.023 | R-FP |
| 37 | -3.62 | 0.002 | R-DLPFC/R-FP |
| 38 | -3.62 | 0.002 | L-DLPFC/L-IFGtri |
| 39 | -2.44 | 0.025 | L-DLPFC/L-FP |
| 41 | -2.40 | 0.028 | R-DLPFC/R-FP |
| 42 | -2.79 | 0.011 | R-IFGtri/R-DLPFC |
| 43 | -2.95 | 0.009 | L-DLPFC |
|  |  |  |  |
| **Incorrect > Correct** | |  |  |
| 30 | 4.05 | 0.001 | L-FP |
| 34 | 3.49 | 0.003 | L-DLPFC/L-FP |
| 35 | 4.04 | 0.001 | L-FP |
| 38 | 2.71 | 0.012 | L-DLPFC/L-IFGtri |
| 39 | 3.32 | 0.003 | L-DLPFC/L-FP |
| 43 | 2.84 | 0.008 | L-DLPFC |

Note: Ch: Channels; L: left; R: right; DLPFC: dorsolateral prefrontal cortex; FP: frontal pole; IFGtri: triangular part of inferior frontal gyrus; IFGoper: opercular part of inferior frontal gyrus; VFR: ventral frontal region.
