# Supplementary material for: Functional reorganization of brain regions supporting artificial grammar learning across the first half year of life
Source: PLoS Biol. 2024 Oct 22;22(10):e3002610. doi: 10.1371/journal.pbio.3002610 (PMC11495551; doi:10.1371/journal.pbio.3002610)
Supplement: S2 Table — (DOCX) [file pbio.3002610.s008.docx]

**S2 Table.** Statistical results of significant paired permutation t-tests under each contrast: Correct vs baseline, Incorrect vs baseline, and Incorrect vs Correct conditions for ΔHbR in Experiment 1.

|  | **Correct > baseline** | | **Incorrect > baseline** | | **Incorrect > Correct** | |
| --- | --- | --- | --- | --- | --- | --- |
| Ch | *t* | *p* | *t* | *p* | *t* | *p* |
| 1 | 1.24 | 0.231 | -0.30 | 0.764 | -1.00 | 0.339 |
| 2 | 1.58 | 0.130 | -1.23 | 0.235 | -2.07 | 0.051 |
| 3 | 1.72 | 0.105 | -0.14 | 0.893 | -1.61 | 0.121 |
| 4 | 0.26 | 0.794 | -0.33 | 0.745 | -0.44 | 0.665 |
| 5 | 0.67 | 0.507 | -0.78 | 0.458 | -1.01 | 0.347 |
| 6 | 1.08 | 0.294 | 0.40 | 0.689 | -0.67 | 0.506 |
| 7 | -0.71 | 0.476 | -0.04 | 0.966 | 0.37 | 0.716 |
| 8 | 0.41 | 0.688 | -0.46 | 0.645 | -0.63 | 0.536 |
| 9 | 0.53 | 0.601 | -0.61 | 0.547 | -0.78 | 0.450 |
| 10 | 0.02 | 0.988 | -0.49 | 0.635 | -0.30 | 0.785 |
| 11 | 0.70 | 0.546 | 0.57 | 0.604 | -0.42 | 0.683 |
| 12 | 1.04 | 0.318 | -1.07 | 0.303 | -1.47 | 0.157 |
| 13 | 0.63 | 0.561 | 0.35 | 0.741 | -0.28 | 0.807 |
| 14 | -0.44 | 0.675 | 0.02 | 0.986 | 0.32 | 0.754 |
| 15 | -0.53 | 0.606 | 0.46 | 0.652 | 0.60 | 0.555 |
| 16 | -0.04 | 0.968 | -0.51 | 0.615 | -0.23 | 0.841 |
| 17 | -0.21 | 0.837 | -0.61 | 0.550 | -0.31 | 0.761 |
| 18 | -0.97 | 0.344 | -0.13 | 0.901 | 0.67 | 0.509 |
| 19 | -0.09 | 0.928 | 0.80 | 0.428 | 0.52 | 0.613 |
| 20 | -0.37 | 0.726 | -0.37 | 0.719 | 0.10 | 0.921 |
| 21 | 0.08 | 0.936 | 0.02 | 0.982 | -0.02 | 0.986 |
| 22 | -0.13 | 0.898 | 0.80 | 0.434 | 0.55 | 0.582 |
| 23 | -0.09 | 0.931 | -0.81 | 0.434 | -0.39 | 0.698 |
| 24 | 0.01 | 0.993 | -0.25 | 0.811 | -0.15 | 0.885 |
| 25 | -0.10 | 0.925 | -1.65 | 0.114 | -0.79 | 0.439 |
| 26 | -0.38 | 0.700 | -1.16 | 0.259 | -0.44 | 0.672 |
| 27 | -0.14 | 0.897 | -0.31 | 0.785 | -0.11 | 0.946 |
| 28 | 0.21 | 0.838 | -0.62 | 0.539 | -0.56 | 0.583 |
| 29 | 0.37 | 0.713 | -2.07 | 0.050 | -1.54 | 0.138 |
| 30 | -0.41 | 0.683 | -0.67 | 0.505 | -0.06 | 0.950 |
| 31 | -0.57 | 0.579 | -0.52 | 0.621 | 0.05 | 0.959 |
| 32 | -0.32 | 0.752 | -1.59 | 0.126 | -0.69 | 0.509 |
| 33 | 1.60 | 0.123 | -0.51 | 0.615 | -1.63 | 0.116 |
| 34 | 0.06 | 0.953 | -0.95 | 0.352 | -0.64 | 0.525 |
| 35 | -0.51 | 0.616 | 0.25 | 0.810 | 0.60 | 0.553 |
| 36 | -0.51 | 0.620 | -1.04 | 0.323 | -0.27 | 0.786 |
| 37 | 1.00 | 0.323 | -0.67 | 0.508 | -1.10 | 0.287 |
| 38 | 0.23 | 0.813 | -1.01 | 0.323 | -0.79 | 0.439 |
| 39 | -0.35 | 0.731 | -0.12 | 0.907 | 0.19 | 0.857 |
| 40 | 0.11 | 0.923 | 0.21 | 0.857 | 0.25 | 0.821 |
| 41 | 0.18 | 0.853 | -0.46 | 0.654 | -0.42 | 0.683 |
| 42 | 0.31 | 0.765 | -0.95 | 0.357 | -0.90 | 0.388 |
| 43 | -0.68 | 0.500 | -0.86 | 0.397 | -0.13 | 0.901 |
| 44 | -0.70 | 0.488 | -0.70 | 0.503 | -0.34 | 0.783 |
| 45 | -0.14 | 0.889 | -0.26 | 0.802 | -0.06 | 0.955 |
| 46 | -0.73 | 0.490 | -0.57 | 0.569 | 0.26 | 0.799 |

Note: Ch: Channels
