# Supplementary material for: Functional reorganization of brain regions supporting artificial grammar learning across the first half year of life
Source: PLoS Biol. 2024 Oct 22;22(10):e3002610. doi: 10.1371/journal.pbio.3002610 (PMC11495551; doi:10.1371/journal.pbio.3002610)
Supplement: S3 Table — (DOCX) [file pbio.3002610.s009.docx]

**S3 Table.** Statistical results of significant paired permutation t-tests under each contrast: Correct vs baseline, Incorrect vs baseline, and Incorrect vs Correct conditions for ΔHbO in Experiment 2.

| Ch | *t* | *p* | Brain region |
| --- | --- | --- | --- |
| **Correct > baseline** | | |  |
| 4 | -3.71 | 0.002 | L-SMG |
| 23 | -3.13 | 0.003 | R-SMG |
|  |  |  |  |
| **Incorrect > baseline** | | |  |
| 4 | 2.32 | 0.031 | L-SMG |
| 11 | 2.17 | 0.045 | L-IFGtri |
| 13 | 2.75 | 0.013 | L-STG |
|  |  |  |  |
| **Incorrect > Correct** | | |  |
| 4 | 4.41 | 0.001 | L-SMG |
| 6 | 2.95 | 0.011 | L-IFGtri |
| 17 | 2.28 | 0.024 | L-STG/L-MTG |
| 23 | 2.25 | 0.046 | R-SMG |
| 29 | 2.25 | 0.040 | R-PreCG/R-IFGoper |

Note: Ch: Channels; L: left; R: right; IFGtri: triangular part of inferior frontal gyrus; IFGoper: opercular part of inferior frontal gyrus; MTG: middle temporal gyrus; PreCG: Precentral gyrus; SMG: supramarginal gyrus; STG: superior temporal gyrus.
