# Supplementary material for: Functional reorganization of brain regions supporting artificial grammar learning across the first half year of life
Source: PLoS Biol. 2024 Oct 22;22(10):e3002610. doi: 10.1371/journal.pbio.3002610 (PMC11495551; doi:10.1371/journal.pbio.3002610)
Supplement: S4 Table — (DOCX) [file pbio.3002610.s010.docx]

**S4 Table.** Statistical results of significant paired permutation t-tests under each contrast: Correct vs baseline, Incorrect vs baseline, and Incorrect vs Correct conditions for ΔHbR in Experiment 2.

|  | **Correct > baseline** | | **Incorrect > baseline** | | **Incorrect > Correct** | |
| --- | --- | --- | --- | --- | --- | --- |
| Ch | *t* | *p* | *t* | *p* | *t* | *p* |
| 1 | 0.18 | 0.856 | -0.61 | 0.560 | -0.60 | 0.551 |
| 2 | **1.89** | **0.032** | -0.83 | 0.457 | **-2.49** | **0.017** |
| 3 | 0.99 | 0.355 | -0.20 | 0.929 | -1.36 | 0.204 |
| 4 | -1.86 | 0.084 | **2.17** | **0.046** | **2.68** | **0.019** |
| 5 | -0.03 | 0.975 | 0.77 | 0.479 | 0.81 | 0.432 |
| 6 | 1.38 | 0.189 | -0.93 | 0.362 | -1.33 | 0.203 |
| 7 | 1.22 | 0.264 | 1.21 | 0.252 | 0.11 | 0.920 |
| 8 | 0.10 | 0.924 | -0.48 | 0.693 | -0.44 | 0.678 |
| 9 | -0.24 | 0.817 | 0.35 | 0.728 | 0.43 | 0.686 |
| 10 | 1.60 | 0.131 | -1.42 | 0.174 | -2.03 | 0.056 |
| 11 | 1.49 | 0.159 | -2.02 | 0.057 | **-2.51** | **0.020** |
| 12 | -0.46 | 0.659 | -1.46 | 0.157 | -0.90 | 0.405 |
| 13 | 0.74 | 0.475 | -0.44 | 0.726 | -0.84 | 0.494 |
| 14 | 1.25 | 0.227 | 1.58 | 0.134 | 0.34 | 0.741 |
| 15 | 1.17 | 0.255 | -1.00 | 0.324 | -1.42 | 0.171 |
| 16 | -0.58 | 0.572 | **-2.16** | **0.044** | -1.16 | 0.261 |
| 17 | 0.48 | 0.636 | 0.72 | 0.507 | 0.20 | 0.860 |
| 18 | -0.11 | 0.911 | -0.71 | 0.536 | -0.46 | 0.660 |
| 19 | 0.07 | 0.948 | 1.20 | 0.264 | 0.84 | 0.415 |
| 20 | -1.25 | 0.237 | -1.97 | 0.062 | -0.02 | 0.981 |
| 21 | -0.89 | 0.400 | -2.01 | 0.062 | -0.32 | 0.769 |
| 22 | 1.25 | 0.227 | 0.04 | 0.967 | -0.77 | 0.449 |
| 23 | -0.02 | 0.990 | 1.76 | 0.102 | 1.56 | 0.143 |
| 24 | -0.07 | 0.949 | 1.06 | 0.313 | 0.83 | 0.424 |
| 25 | 0.35 | 0.757 | -0.85 | 0.419 | -1.13 | 0.287 |
| 26 | -0.17 | 0.874 | 1.10 | 0.326 | 0.70 | 0.517 |
| 27 | -0.78 | 0.448 | -0.56 | 0.574 | 0.01 | 0.989 |
| 28 | -1.09 | 0.315 | 1.09 | 0.301 | 1.92 | 0.078 |
| 29 | -1.18 | 0.257 | -0.15 | 0.886 | 0.51 | 0.618 |
| 30 | 0.38 | 0.715 | 1.74 | 0.090 | 1.05 | 0.310 |
| 31 | 0.61 | 0.535 | 0.23 | 0.835 | -0.24 | 0.818 |
| 32 | 0.08 | 0.939 | -1.85 | 0.081 | -1.62 | 0.124 |
| 33 | 0.76 | 0.464 | -0.29 | 0.773 | -0.71 | 0.482 |
| 34 | -0.16 | 0.876 | 0.97 | 0.344 | 0.93 | 0.379 |
| 35 | -0.40 | 0.691 | 0.49 | 0.628 | 0.62 | 0.544 |
| 36 | 0.57 | 0.571 | -0.33 | 0.744 | -0.62 | 0.545 |
| 37 | 0.84 | 0.412 | -1.16 | 0.264 | -1.42 | 0.173 |
| 38 | -1.02 | 0.320 | -0.98 | 0.354 | -0.09 | 0.944 |
| 39 | -0.47 | 0.676 | **-2.40** | **0.028** | -0.69 | 0.506 |
| 40 | 0.44 | 0.669 | -1.32 | 0.210 | -1.23 | 0.239 |
| 41 | 0.75 | 0.495 | **-2.33** | **0.032** | **-2.10** | **0.047** |
| 42 | 0.32 | 0.768 | -0.62 | 0.545 | -0.57 | 0.597 |
| 43 | -1.64 | 0.109 | -1.29 | 0.209 | 0.42 | 0.699 |
| 44 | -0.98 | 0.338 | -2.07 | 0.056 | -0.72 | 0.495 |

Note: Ch: Channels; Significant results are indicated in bold.
