# Supplementary material for: Functional reorganization of brain regions supporting artificial grammar learning across the first half year of life
Source: PLoS Biol. 2024 Oct 22;22(10):e3002610. doi: 10.1371/journal.pbio.3002610 (PMC11495551; doi:10.1371/journal.pbio.3002610)
Supplement: S5 Table — (DOCX) [file pbio.3002610.s011.docx]

**S5 Table.** Base frequency of pitch-shifted variants for acoustic categories A, B, C, and D

| Pitch contour | Category | Low frequency (Hz) | | | | | High frequency (Hz) | | | | |
| --- | --- | --- | --- | --- | --- | --- | --- | --- | --- | --- | --- |
|  |  | 1 | 2 | 3 | 4 | 5 | 6 | 7 | 8 | 9 | 10 |
| Rising | A | 500 | 550 | 600 | 650 | 700 | 900 | 950 | 1000 | 1050 | 1100 |
| Slow wave | B | 500 | 550 | 600 | 650 | 700 | 900 | 950 | 1000 | 1050 | 1100 |
| Fast wave | C | 500 | 550 | 600 | 650 | 700 | 900 | 950 | 1000 | 1050 | 1100 |
| Falling | D | 500 | 550 | 600 | 650 | 700 | 900 | 950 | 1000 | 1050 | 1100 |
