# Supplementary material for: Functional reorganization of brain regions supporting artificial grammar learning across the first half year of life
Source: PLoS Biol. 2024 Oct 22;22(10):e3002610. doi: 10.1371/journal.pbio.3002610 (PMC11495551; doi:10.1371/journal.pbio.3002610)
Supplement: S6 Table — (DOCX) [file pbio.3002610.s012.docx]

**S6 Table.** Base frequency of pitch-shifted variants for acoustic categories X1 and X2

| Pitch contour | Category | Low frequency (Hz) | | | | | | | |
| --- | --- | --- | --- | --- | --- | --- | --- | --- | --- |
|  |  | 1 | 2 | 3 | 4 | 5 | 6 | 7 | 8 |
| Big spike | X1 | 500 | 530 | 560 | 590 | 620 | 650 | 680 | 710 |
| Small spike | X2 | 500 | 530 | 560 | 590 | 620 | 650 | 680 | 710 |
| Pitch contour | Category | High frequency (Hz) | | | | | | | |
|  |  | 9 | 10 | 11 | 12 | 13 | 14 | 15 | 16 |
| Big spike | X1 | 900 | 930 | 960 | 990 | 1120 | 1150 | 1180 | 1110 |
| Small spike | X2 | 900 | 930 | 960 | 990 | 1120 | 1150 | 1180 | 1110 |
