# Supplementary material for: Functional reorganization of brain regions supporting artificial grammar learning across the first half year of life
Source: PLoS Biol. 2024 Oct 22;22(10):e3002610. doi: 10.1371/journal.pbio.3002610 (PMC11495551; doi:10.1371/journal.pbio.3002610)
Supplement: S7 Table — (DOCX) [file pbio.3002610.s013.docx]

**S7 Table.** Stimulus lists used in Experiments 1 and 2

|  | List 1 | List 2 | List 3 | List 4 |
| --- | --- | --- | --- | --- |
| Standard triplets | A_1-5_ X_1-8_ B_1-5_  C_1-5_ X_1-8_ D_1-5_ | A_1-5_ X_1-8_ D_1-5_  C_1-5_ X_1-8_ B_1-5_ | A_6-10_ X_9-16_ B_6-10_  C_6-10_ X_9-16_ D_6-10_ | A_6-10_ X_9-16_ D_6-10_  C_6-10_ X_9-16_ B_6-10_ |
| Correct triplets | A_6-10_ X_11-16_ B_6-10_  C_6-10_ X_11-16_ D_6-10_ | A_6-10_ X_11-16_ D_6-10_  C_6-10_ X_11-16_ B_6-10_ | A_1-5_ X_1-6_ B_1-5_  C_1-5_ X_1-6_ D_1-5_ | A_1-5_ X_1-6_ D_1-5_  C_1-5_ X_1-6_ B_1-5_ |
| Incorrect triplets | A_6-10_ X_11-16_ D_6-10_  C_6-10_ X_11-16_ B_6-10_ | A_6-10_ X_11-16_ B_6-10_  C_6-10_ X_11-16_ D_6-10_ | A_1-5_ X_1-6_ D_1-5_  C_1-5_ X_1-6_ B_1-5_ | A_1-5_ X_1-6_ B_1-5_  C_1-5_ X_1-6_ D_1-5_ |

Note: X represents X1 or X2. Numbers indicate the pitch variant of each acoustic category.
